# Profiles of neuropsychiatric toxicity associated with different endocrine therapies for breast cancer: a global pharmacovigilance study based on FAERS and VigiAccess

**DOI:** 10.3389/fphar.2026.1731849

**Published:** 2026-03-05

**Authors:** Guoqiang Li, Mengqi Yang, Lei Zhang, Xin Li, Xiaoliang Wu, Feng Peng, Yajie Liu

**Affiliations:** Department of Radiation Oncology, Peking University Shenzhen Hospital, Hong Kong University of Science and Technology Medical Center, Shenzhen, Guangdong, China

**Keywords:** breast cancer, endocrine therapy, neurotoxicity, psychiatric disorders, real-world

## Abstract

**Purpose:**

Breast cancer is the most common malignancy in women. Hormone receptor-positive, human epidermal growth factor receptor 2-negative (HR+/HER2-) breast cancer patients rely on endocrine therapy as their fundamental systemic treatment strategy. This study aims to comprehensively evaluate the patterns of neurotoxicity and psychotoxicity across different classes of endocrine therapy.

**Methods:**

Pharmacovigilance data related to endocrine therapy for breast cancer from the FDA Adverse Event Reporting System (FAERS) and WHO VigiAccess database were utilized. The disproportionality algorithms, including reporting odds ratio and information component, were employed in FAERS to investigate the patterns, influencing factors, and outcomes of neurological and psychiatric event burdens in selective estrogen receptor modulators (SERMs), selective estrogen receptor degraders (SERDs), and aromatase inhibitors (AIs). Sensitivity analysis was conducted using VigiAccess as a supplementary data source.

**Results:**

A total of 64,731 FAERS and 116,605 VigiAccess safety reports on endocrine therapies were analyzed. Neurotoxic and psychiatric events accounted for approximately 20% and 10% of these reports, respectively. The most common neurologic adverse events were headache, dizziness, and sensory impairment, while insomnia, depression, and anxiety were the most frequent psychiatric events. The disproportionality analysis indicated that SERMs showed several strong neurovascular safety signals, such as cerebral venous thrombosis and dural arteriovenous fistula. Both SERMs and AIs showed positive signals for depression, whereas SERDs did not. All therapies exhibited an “early failure” pattern in time-to-onset analyses (β = 0.54–0.66).

**Conclusion:**

This study conducted a comprehensive pharmacovigilance assessment of neurotoxicity and psychiatric events in endocrine therapy for breast cancer. The findings indicated heterogeneous patterns of neuropsychiatric safety signals and reporting burdens across drug classes, offering new insights relevant to clinical monitoring practices.

## Introduction

Breast cancer is the most commonly occurring cancer and the leading cause of cancer-related death among women worldwide (Bray et al., 2024). Approximately 70% of breast cancer cases are hormone receptor-positive (HR+) and human epidermal growth factor receptor 2-negative (HER2-). For these patients, endocrine therapy has become a cornerstone of systematic treatment, significantly reducing recurrence risk and improving long-term survival (Waks and Winer, 2019; Huppert et al., 2023). Depending on their mechanisms of action, the most commonly used endocrine therapies include selective estrogen receptor modulators (SERMs), selective estrogen receptor degraders (SERDs), and aromatase inhibitors (AIs). These agents suppress estrogen-dependent tumor progression through distinct molecular pathways (Patel et al., 2023).

With advances in early detection and precision therapy, breast cancer survival has improved substantially in recent years. However, the prolonged use of endocrine therapy has drawn increasing attention to treatment-related adverse effects, which may impair quality of life and lead to premature therapy discontinuation, potentially compromising clinical benefit (Patel et al., 2023). Among these adverse effects, neurotoxicity and psychiatric toxicity have become areas of growing clinical concern (Haggstrom et al., 2022). Neurological and psychiatric symptoms are frequently observed in patients with breast cancer, such as memory decline, cognitive impairment, and depression (Kim et al., 2015; Tsaras et al., 2018; Pilevarzadeh et al., 2019; Lee Meeuw Kjoe et al., 2023; Piffoux et al., 2024). Nevertheless, the association between endocrine therapies and these symptoms remains unclear, particularly in terms of differences across drug classes. This is likely due to their often subtle and gradual onset, as well as the complexity of clinical recognition and diagnosis.

Previous evidence suggests that tamoxifen can cross the blood-brain barrier and disrupt neuronal function by directly or indirectly affecting dopaminergic and other neurotransmitter systems (Chen et al., 2002; 2014; Denk et al., 2015; Novick et al., 2020). Estrogen receptors are present in several brain regions, including the hippocampus, where they may interact with estrogen receptor antagonists (Almey et al., 2015). However, some studies have reported that tamoxifen may exhibit both antagonistic and agonistic properties in the brain, depending on the level of endogenous estrogen (Azizi-Malekabadi et al., 2015; Pandey et al., 2016; Novick et al., 2020). As a result, the neurological impact of endocrine therapy in breast cancer remains highly uncertain. Furthermore, for newer agents such as elacestrant, safety data are still limited due to their recent market approval, necessitating reliance on real-world data sources for early signal detection of potential adverse events. Large-scale spontaneous reporting systems offer a feasible approach to systematically profiling neuropsychiatric adverse events associated with endocrine therapies. The U.S. Food and Drug Administration Adverse Event Reporting System (FAERS), one of the largest pharmacovigilance databases globally, contains millions of real-world adverse drug event (ADE) reports submitted by healthcare professionals, patients, and pharmaceutical companies, which is widely used in signal detection, drug re-evaluation, and pharmacovigilance research. By leveraging large pharmacovigilance databases, it is possible to detect potential drug-related adverse events and to estimate the statistical strength of drug-event associations through quantitative disproportionality analysis (Beninger, 2018).

In this study, we conducted a systematic pharmacovigilance analysis using data from FAERS and the WHO’s VigiAccess databases to investigate neurotoxic and psychiatric adverse events associated with three major classes of endocrine therapies (SERMs, SERDs, and AIs). We applied pharmacovigilance algorithms to identify statistically significant safety signals and characterized toxicity profiles across endocrine therapies. Our goal is to provide evidence to support personalized treatment strategies, inform long-term patient management, and enhance awareness of potential neuropsychiatric risks associated with endocrine therapies.

## Methods

### Study design

This was an observational pharmacovigilance study based on individual case safety reports (ICSRs) from the FAERS and aggregate-level data from the WHO’s VigiAccess. Disproportionality analysis and descriptive statistical methods were used to identify potential safety signals and characterize neurotoxic and psychiatric adverse events associated with endocrine therapies in breast cancer. The study followed the 2024 guidelines for Reporting of a Disproportionality Analysis for Drug Safety Signal Detection Using Individual Case Safety Reports in Pharmacovigilance to ensure reporting transparency (Fusaroli et al., 2024).

### Data sources

The primary data source was the FAERS database, covering the period from Q1 2004 (the earliest quarter available in FAERS) to Q4 2024. FAERS is one of the most representative global pharmacovigilance databases, compiling voluntary reports of post-marketing adverse drug events (ADEs). All ADEs were re-coded using the Medical Dictionary for Regulatory Activities (MedDRA, version 26.1). We extracted all available ICSRs from the defined period. Duplicate entries were removed by retaining only the version with the highest version number for each case.

In addition, we accessed the WHO’s VigiAccess platform to supplement safety data on endocrine therapies used in breast cancer (https://www.vigiaccess.org/) (Liang et al., 2024; Lakhani et al., 2025). VigiAccess is a web-based tool that allows users to retrieve aggregated statistics on potential adverse drug reactions from VigiBase, the WHO global database of reported adverse events of medicinal products, which includes approximately 40 million safety records from over 180 member countries or regions of the WHO Programme for International Drug Monitoring (PIDM). Data were accessed on 13 May 2025, without restrictions on start date or other any filter.

### Drugs and events of interest

The drugs of interest included tamoxifen, toremifene, fulvestrant, elacestrant, anastrozole, letrozole, and exemestane, which are the most commonly used endocrine therapies for breast cancer. No restrictions were applied regarding age, sex, or country of origin. In addition to generic names, we searched for relevant trade and synonym names (such as Soltamox, Fareston, Faslodex). Drugs were categorized based on their mechanisms of action into three groups. Specifically, tamoxifen and toremifene were classified as selective estrogen receptor modulators (SERMs); fulvestrant and elacestrant as selective estrogen receptor degraders (SERDs); and anastrozole, letrozole, and exemestane as aromatase inhibitors (AIs). Drugs were grouped by active ingredient, regardless of brand or salt form.

Adverse events of interest were defined as all events coded under the Nervous system disorders and Psychiatric disorders of MedDRA primary System Organ Classes (SOCs). In VigiAccess, drugs and adverse events were standardized using WHODrug and MedDRA terminology, respectively. We extracted all ADRs reported under the above two SOCs associated with the seven drugs and performed descriptive analyses.

### Statistical methods

Descriptive statistics were used to summarize the number and proportion of neuropsychiatric ADRs reported for each drug category during the study period. For signal detection, two disproportionality methods were applied: the Reporting Odds Ratio (ROR) and the Information Component (IC) methods (Shi et al., 2023; Zhu et al., 2024).

$$\text{ROR}=\frac{\left(a/c\right)}{\left(b/d\right)}=\frac{a\times d}{b\times c}$$

$${\text{IC}=\log}_{2}\frac{p\left(x,y\right)}{p\left(x\right)p\left(y\right)}=\log_{2}\frac{a\left(a+b+c+d\right)}{\left(a+b\right)\left(a+c\right)}$$

Where *a* = number of reports of the event with the drug of interest; *b* = number of reports without the event with the drug of interest; *c* = number of reports of the event with other drugs; and *d* = number of reports without the event with other drugs.

A positive signal was defined only if both criteria were met: the lower bound of the 95% confidence interval for ROR >1, and the lower bound of the IC (IC025) > 0. Additionally, each signal required at least three reports. A positive signal suggests a potential statistical association between a drug and a specific adverse event. Given that the ROR serves as the pharmacovigilance equivalent of the odds ratio and is easy to interpret, signal strength was classified based on ROR magnitude: very strong (ROR >30, indicating the signal event was reported at least 30 times more frequently for the drug being analyzed compared to other drugs); strong (ROR between 5 and 30, indicating the signal event was reported 5 to 30 times more frequently for the drug being analyzed compared to other drugs); and weak (ROR <5, indicating the signal event was reported more frequently for the drug being analyzed compared to other drugs, but less than five times more frequently).

Because VigiAccess does not provide individual-level reports, we conducted descriptive analyses of ADR counts and proportions at the Preferred Term and High-Level Term levels as supplementary sensitivity analyses. Logistic regression models were used to evaluate disproportionality reporting across endocrine therapy classes, adjusting for age, sex, reporting year, reporter qualification, reporting country, and concurrent ovarian function suppression (OFS) therapy (leuprolide, goserelin, triptorelin). Time-to-onset (TTO) distributions were analyzed using the Weibull shape parameter test. The scale parameter (α), shape parameter (β), median time, and interquartile range were used to describe TTO patterns. Smaller α values indicate narrower time distributions, while larger values indicate wider variability. A lower 95% CI bound of β > 1 indicates a *wear-out failure* pattern (increasing hazard over time), whereas an upper bound of β < 1 indicates an *early failure* pattern (decreasing hazard over time). We also used Wilcoxon rank-sum tests to assess differences in TTO among SERM, SERD, and AI classes. Finally, we summarized clinical outcomes for neuropsychiatric ADRs across endocrine therapies using FAERS data. All statistical analyses and data visualizations were performed using RStudio (version 4.3.1).

## Results

### Population characteristics and descriptive analysis

**FIGURE 1:**
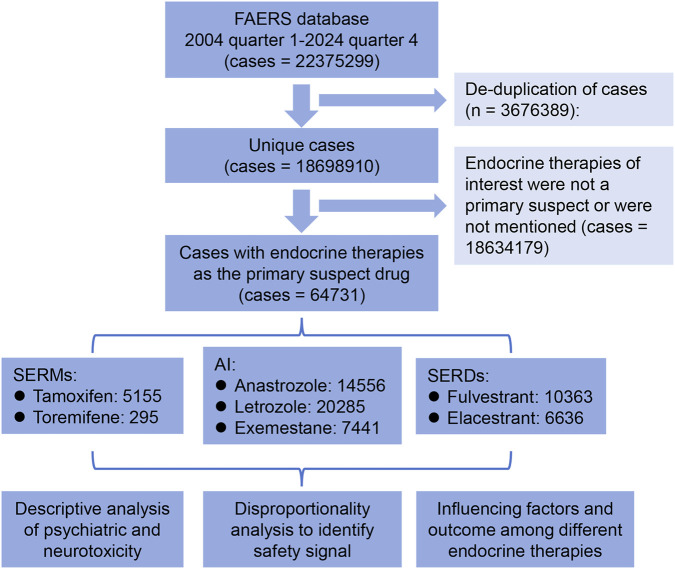
Patient selection flow diagram. SERMs, selective estrogen receptor modulators; SERDs, selective estrogen receptor degraders; AIs, aromatase inhibitors.

From the first quarter of 2004 to the fourth quarter of 2024, a total of 22,375,299 safety reports were retrieved from the FAERS database. After excluding 3,676,389 duplicate reports, 18,698,910 unique cases were retained. Among these, 64,731 cases reported endocrine therapies of interest as the primary suspected drug, including tamoxifen (n = 5,155), toremifene (n = 295), anastrozole (n = 14,556), letrozole (n = 20,285), exemestane (n = 7,441), fulvestrant (n = 10,363), and elacestrant (n = 6,636). The remaining 18,634,179 reports, in which endocrine therapies were not listed as the primary suspect or were unrelated, served as the comparator group for disproportionality analysis of safety signal (Figure 1).

**FIGURE 2:**
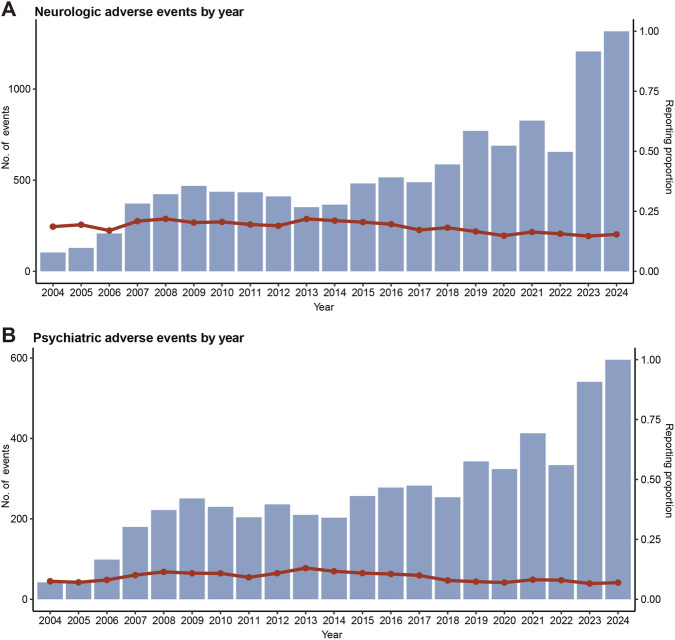
Annual trend distribution of neurotoxic and psychiatric events in breast cancer endocrine therapy in the FAERS database. **(A)** Annual number and proportion of neurologic adverse events from 2004 to 2024; **(B)** Annual number and proportion of psychiatric adverse events from 2004 to 2024. FAERS, Food and Drug Administration Adverse Event Reporting System.

Overall, the majority of included safety cases involved patients aged over 60 years (n = 26,465, 61.65%), and most were female (n = 58,942, 98.25%) (Table 1). Between 2004 and 2024, the absolute number of reported neurologic or psychiatric adverse events steadily increased, although their relative proportions remained stable, fluctuating around 20% and 10%, respectively (Figures 2A,B). The United States accounted for the highest number of reports (n = 29,265, 46.73%), followed by Germany (n = 5,802, 9.26%), the United Kingdom (n = 4,080, 6.51%), France (n = 4,061, 6.48%), and Canada (n = 3,609, 5.76%). The proportion of patients receiving concomitant ovarian function suppression was 3.25%.

**TABLE 1:** Characteristics of adverse reaction reports of different endocrine therapies in the FAERS (up to 2024).

Characteristic	Overall, N = 64,731	Anastrozole, N = 14,556	Elacestrant, N = 6,636	Exemestane, N = 7,441	Fulvestrant, N = 10,363	Letrozole, N = 20,285	Tamoxifen, N = 5,155	Toremifene, N = 295
Age groups (years)								
≤45	3,578 (8.334%)	507 (5.068%)	349 (5.649%)	223 (4.170%)	533 (9.022%)	1,305 (11.02%)	654 (18.77%)	7 (4.321%)
45–60	12,887 (30.02%)	2,863 (28.62%)	1,647 (26.66%)	1,400 (26.18%)	1,948 (32.97%)	3,543 (29.91%)	1,442 (41.38%)	44 (27.16%)
>60	26,465 (61.65%)	6,633 (66.31%)	4,182 (67.69%)	3,725 (69.65%)	3,427 (58.01%)	6,998 (59.07%)	1,389 (39.86%)	111 (68.52%)
Unknown	21,801	4,553	458	2,093	4,455	8,439	1,670	133
Sex								
Female	58,942 (98.25%)	13,428 (97.39%)	6,291 (99.09%)	6,863 (99.15%)	9,267 (99.03%)	18,513 (98.61%)	4,397 (95.44%)	183 (95.81%)
Male	1,047 (1.745%)	360 (2.611%)	58 (0.914%)	59 (0.852%)	91 (0.972%)	261 (1.390%)	210 (4.558%)	8 (4.188%)
Unknown	4,742	768	287	519	1,005	1,511	548	104
Reporting year								
2004–2006	2,450 (3.785%)	616 (4.232%)	0 (0%)	377 (5.067%)	127 (1.226%)	478 (2.356%)	805 (15.62%)	47 (15.93%)
2007–2009	6,028 (9.312%)	3,630 (24.94%)	0 (0%)	559 (7.512%)	277 (2.673%)	858 (4.230%)	674 (13.07%)	30 (10.17%)
2010–2012	6,516 (10.07%)	2,730 (18.76%)	0 (0%)	987 (13.26%)	557 (5.375%)	1,845 (9.095%)	355 (6.887%)	42 (14.24%)
2013–2015	5,699 (8.804%)	1,584 (10.88%)	0 (0%)	1,365 (18.34%)	678 (6.543%)	1,558 (7.681%)	461 (8.943%)	53 (17.97%)
2016–2018	8,703 (13.44%)	1,769 (12.15%)	0 (0%)	1,494 (20.08%)	1,541 (14.87%)	3,046 (15.02%)	810 (15.71%)	43 (14.58%)
2019–2021	14,348 (22.17%)	2,187 (15.02%)	0 (0%)	1,492 (20.05%)	3,142 (30.32%)	6,293 (31.02%)	1,195 (23.18%)	39 (13.22%)
2022–2024	20,987 (32.42%)	2,040 (14.01%)	6,636 (100%)	1,167 (15.68%)	4,041 (38.99%)	6,207 (30.60%)	855 (16.59%)	41 (13.90%)
Reporter type								
Health-professional	34,550 (53.37%)	6,375 (43.80%)	869 (13.10%)	3,848 (51.71%)	6,961 (67.17%)	13,373 (65.93%)	2,983 (57.87%)	141 (47.80%)
Consumer	24,777 (38.28%)	5,940 (40.81%)	5,709 (86.03%)	3,311 (44.50%)	2,186 (21.09%)	6,029 (29.72%)	1,551 (30.09%)	51 (17.29%)
Other/unknown	5,404 (8.348%)	2,241 (15.40%)	58 (0.874%)	282 (3.790%)	1,216 (11.73%)	883 (4.353%)	621 (12.05%)	103 (34.92%)
Country of occurrence								
United States	29,265 (46.73%)	8,422 (59.31%)	6,526 (99.21%)	4,187 (57.50%)	3,055 (29.73%)	5,172 (26.86%)	1,855 (38.85%)	48 (18.25%)
Germany	5,802 (9.264%)	594 (4.183%)	10 (0.152%)	644 (8.844%)	1,322 (12.87%)	3,075 (15.97%)	157 (3.288%)	0 (0%)
United Kingdom	4,080 (6.514%)	945 (6.655%)	3 (0.046%)	257 (3.529%)	390 (3.796%)	1,763 (9.155%)	718 (15.04%)	4 (1.521%)
France	4,061 (6.484%)	721 (5.077%)	18 (0.274%)	428 (5.878%)	478 (4.652%)	1,836 (9.534%)	580 (12.15%)	0 (0%)
Canada	3,609 (5.762%)	595 (4.190%)	0 (0%)	135 (1.854%)	1,103 (10.73%)	1,357 (7.046%)	419 (8.775%)	0 (0%)
Other country	15,814 (25.25%)	2,923 (20.58%)	21 (0.319%)	1,631 (22.40%)	3,927 (38.22%)	6,055 (31.44%)	1,046 (21.91%)	211 (80.23%)
Unknown	2,100	356	58	159	88	1,027	380	32
Concurrent OFS use (yes)	2,104 (3.250%)	250 (1.718%)	83 (1.251%)	152 (2.043%)	305 (2.943%)	1,081 (5.329%)	223 (4.326%)	10 (3.390%)

OSF, ovarian function suppression.

Reports in the WHO’s VigiAccess database were predominantly from Europe and the United States. As of May 2025, the number of adverse event reports associated with each drug was as follows: Tamoxifen (n = 26,160), Toremifene (n = 387), Fulvestrant (n = 14,973), Elacestrant (n = 6,746), Anastrozole (n = 20,440), Letrozole (n = 34,603), and Exemestane (n = 13,296).

### Neurological toxicity profile of endocrine therapy

**FIGURE 3:**
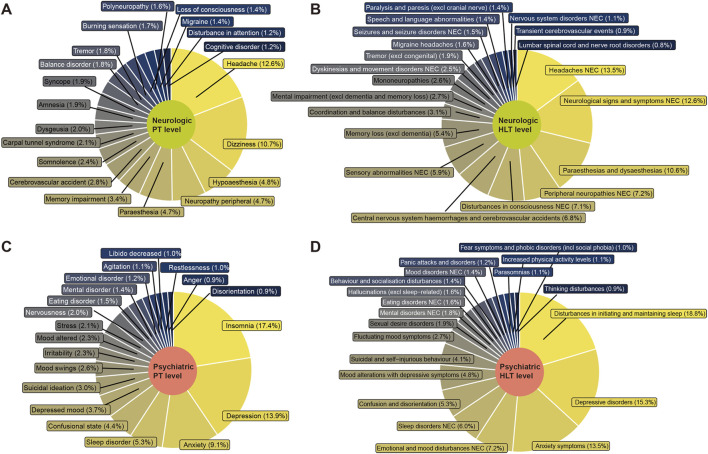
Burden of neurotoxic and psychiatric events at the PT and HLT levels in the FAERS database for breast cancer endocrine therapies. **(A)** Top 20 neurologic events by report proportion at the PT level; **(B)** Top 20 neurologic events by report proportion at the HLT level; **(C)** Top 20 psychiatric events by report proportion at the PT level; **(D)** Top 20 psychiatric events by report proportion at the HLT level. PT, Preferred Term; HLT, High Level Term.

At the Preferred Term (PT) level, the most frequently reported neurological adverse event was headache (12.6%), followed by dizziness (10.7%), hypoaesthesia (4.8%), peripheral neuropathy (4.7%), paraesthesia (4.7%), and memory impairment (3.4%) (Figure 3A). At the High-Level Term (HLT) level, headache (13.5%), neurological signs (12.6%), and paraesthesias and dysaesthesias (10.6%) represented a significant burden (Figure 3B).

Consistently, data extracted from the WHO VigiAccess database confirmed that headache, dizziness, paraesthesia, peripheral neuropathy, and hypoaesthesia were the most frequently reported neurological events across SERMs, AIs, and SERDs (Supplementary Figures 1–3A,B).

### Psychiatric toxicity profile of endocrine therapy

For psychiatric adverse events, insomnia (17.4%), depression (13.9%), and anxiety (9.1%) were the most prominent (Figure 3C). This trend was also reflected at the HLT level, indicating conditions related to sleep, depression, and anxiety (Figure 3D). Data from the WHO VigiAccess database was consistent with these findings. Notably, a relatively higher proportion of depressive disorders was reported with SERMs (20.2%) compared to SERDs (11.3%) and AIs (16.0%) (Supplementary Figures 1–3C,D).

### Neurotoxicity safety signals

**FIGURE 4:**
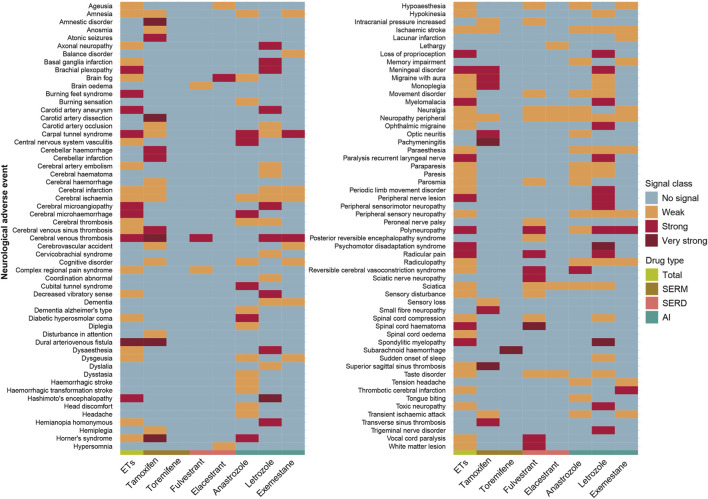
Neurotoxicity safety signals of different breast cancer endocrine therapies. Signal strength was classified by ROR magnitude: very strong (ROR >30); strong (ROR between 5 and 30); and weak (ROR <5). ETs, Endocrine Therapies. SERMs, selective estrogen receptor modulators; SERDs, selective estrogen receptor degraders; AIs, aromatase inhibitors.

We systematically assessed the neurotoxicity safety signals associated with endocrine therapies, both overall and at the level of individual agents. Notable heterogeneity was observed in the signal profiles across drugs (Figure 4). Tamoxifen showed several exceptionally strong neurotoxicity signals, including dural arteriovenous fistula (ROR = 460.33, 95%CI: 245.70–862.44), amnestic disorder (ROR = 32.78, 95%CI: 12.25–87.73), carotid artery dissection (ROR = 36.80, 95%CI: 13.74–98.56), horner’s syndrome (ROR = 30.10, 95%CI: 11.25–80.55), pachymeningitis (ROR = 47.96, 95%CI: 15.35–149.85). Toremifene was notably associated with subarachnoid haemorrhage (ROR = 31.47, 95% CI: 11.78–84.08), while fulvestrant showed a strong signal for spinal cord haematoma (ROR = 57.66, 95% CI: 32.37–102.72). In the case of letrozole, strong signals were detected for Hashimoto’s encephalopathy (ROR = 76.99, 95% CI: 35.24–168.22), psychomotor disadaptation syndrome (ROR = 58.45, 95% CI: 17.98–190.05), and spondylitic myelopathy (ROR = 41.33, 95% CI: 15.06–113.39) (Supplementary Table 1).

Among the five most frequently reported neurological adverse events, headache was overreported only among users of anastrozole (ROR = 1.17, 95% CI: 1.08–1.26). Dizziness, despite its frequency, did not constitute a signal for any agent. Hypoaesthesia was disproportionately reported for anastrozole, exemestane, and fulvestrant; paraesthesia for anastrozole, exemestane, and letrozole; and peripheral neuropathy for all endocrine therapies except toremifene (Supplementary Table 1).

### Psychiatric safety signals

**FIGURE 5:**
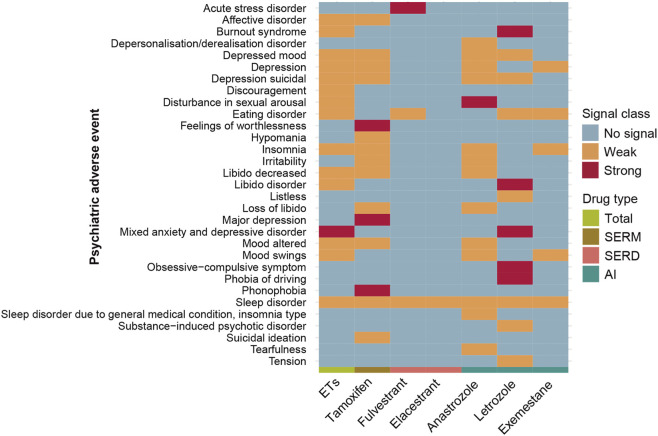
Psychiatric safety signals of different breast cancer endocrine therapies. Signal strength was classified by ROR magnitude: very strong (ROR >30); strong (ROR between 5 and 30); and weak (ROR <5). ETs, Endocrine Therapies. SERMs, selective estrogen receptor modulators; SERDs, selective estrogen receptor degraders; AIs, aromatase inhibitors.

Endocrine therapies also exhibited varied psychiatric safety signal profiles across different agents (Figure 5). Mixed anxiety and depressive disorder emerged as a notable signal across the class (ROR = 8.62, 95% CI: 4.43–16.76). For tamoxifen, phonophobia, feelings of worthlessness, and major depression were identified as strong psychiatric signals. Fulvestrant was associated with acute stress disorder, while anastrozole showed a signal for disturbance in sexual arousal. Letrozole was linked to several strong psychiatric signals, including mixed anxiety and depressive disorder, phobia of driving, libido disorder, burnout syndrome, and obsessive-compulsive symptoms (Figure 5; Supplementary Table 2).

Among the most commonly reported psychiatric adverse events, both insomnia and depression were disproportionately reported with anastrozole, exemestane, and tamoxifen.

### Disproportionality analysis across endocrine therapy types

**FIGURE 6:**
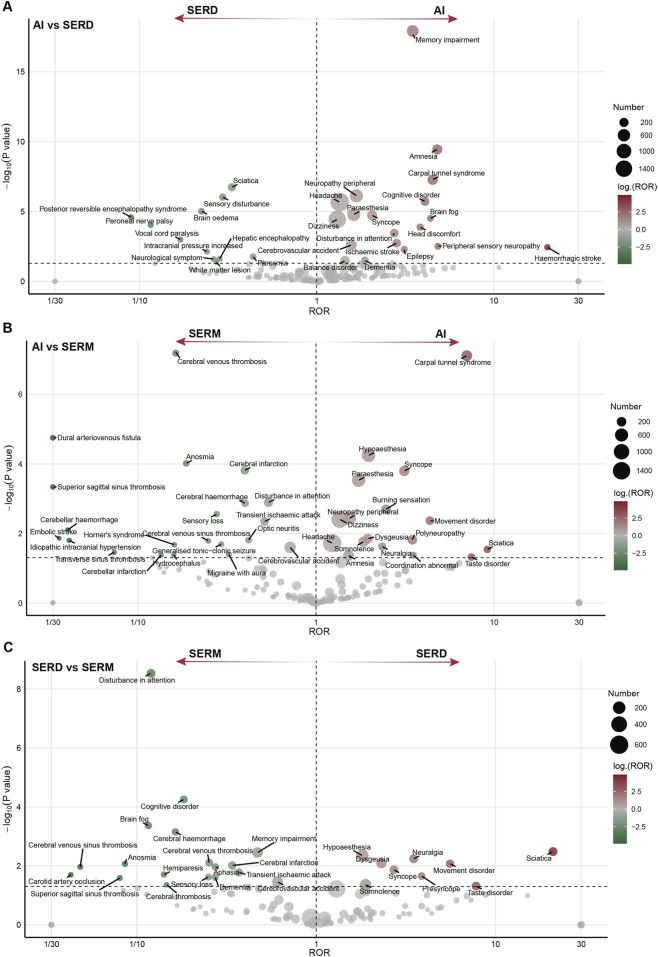
Disproportionate reporting differences of neurologic events across different endocrine therapy classes. **(A)** SERDs vs. AIs; **(B)** SERMs vs. AIs; **(C)** SERMs vs. SERDs. Multivariable logistic regression, adjusted for age, sex, reporting year, reporter qualification, reporting country, and concurrent ovarian function suppression, was used for analysis. SERMs, selective estrogen receptor modulators; SERDs, selective estrogen receptor degraders; AIs, aromatase inhibitors; ROR, Reporting Odds Ratio.

**FIGURE 7:**
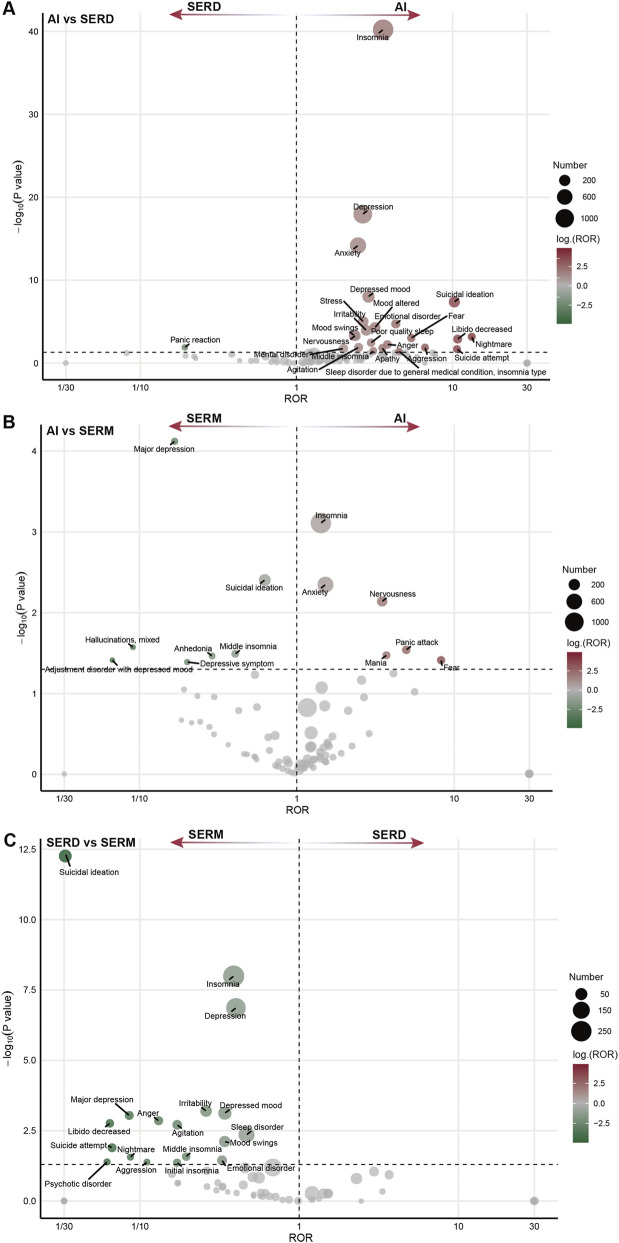
Disproportionate reporting differences of psychiatric events across different endocrine therapy classes. **(A)** SERDs vs. AIs; **(B)** SERMs vs. AIs; **(C)** SERMs vs. SERDs. Multivariable logistic regression, adjusted for age, sex, reporting year, reporter qualification, reporting country, and concurrent ovarian function suppression, was used for analysis. SERMs, selective estrogen receptor modulators; SERDs, selective estrogen receptor degraders; AIs, aromatase inhibitors; ROR, Reporting Odds Ratio.

After adjusting for age, sex, year of report, reporter occupation, country, and the use of OFS, we compared the reporting patterns of neuropsychiatric adverse events across different classes of endocrine therapy. Notable differences in reporting patterns were observed across the three major drug classes. For neurological events, AIs showed higher reporting frequencies of carpal tunnel syndrome, peripheral neuropathy, headache, and paraesthesia compared to SERDs and SERMs. In contrast, reports of cerebral venous thrombosis, anosmia, cerebral infarction, superior sagittal sinus thrombosis, and cerebral haemorrhage were more frequently observed with SERM use. Additionally, sciatica was more commonly reported in association with SERDs (Figures 6A–C). Similarly, distinct psychiatric profiles were observed across endocrine therapy classes. Depression-related events were more frequently reported with SERMs, whereas insomnia and anxiety were more commonly reported with AIs (Figures 7A–C).

### Potential risk factors and stratified analysis of neurological and psychiatric events in endocrine therapy

To further explore factors associated with neuropsychiatric toxicity, we performed both univariate and multivariate logistic regression analyses. Interestingly, among users of AIs, both younger patients (≤45 years) and older patients (>60 years) showed significantly lower reporting proportion of neurotoxicity compared to those aged 45–60 years. The adjusted RORs were 0.86 (95% CI: 0.75–0.99) and 0.91 (95% CI: 0.85–0.97), respectively. No significant age-related differences were observed for neurotoxicity in users of SERDs or SERMs (Table 2). However, for psychiatric toxicity, older patients (>60 years) receiving AIs and SERMs reported less reporting proportion, with adjusted ORs of 0.81 (95% CI: 0.75–0.89) and 0.58 (95% CI: 0.43–0.77), respectively. Notably, OFS use did not significantly influence the reporting frequency of either neurological or psychiatric events for any endocrine therapy (Table 2).

**TABLE 2:** Disproportionality analysis of age and OSF use status for neurologic and psychotropic toxicity among different endocrine therapies.

Groups/model	Age group (years)			OSF use status	
	45–60	≤45	>60	No	Yes
Neurological disorders		OR (95%CI)		OR (95%CI)	
AI					
Univariable	Ref	0.74 (0.65–0.84)	0.94 (0.88–1.00)	Ref	0.75 (0.64–0.86)
Multivariable	Ref	0.86 (0.75–0.99)	0.91 (0.85–0.97)	Ref	0.86 (0.73–1.00)
SERDs					
Univariable	Ref	0.85 (0.67–1.06)	1.12 (1.00–1.26)	Ref	0.95 (0.70–1.26)
Multivariable	Ref	0.88 (0.69–1.11)	1.09 (0.97–1.23)	Ref	1.18 (0.85–1.60)
SERMs					
Univariable	Ref	0.97 (0.76–1.23)	0.85 (0.70–1.03)	Ref	0.75 (0.50–1.10)
Multivariable	Ref	0.99 (0.78–1.27)	0.88 (0.72–1.07)	Ref	0.82 (0.54–1.21)

Psychiatric disorders		OR (95%CI)		OR (95%CI)	
AI					
Univariable	Ref	0.92 (0.78–1.07)	0.86 (0.79–0.93)	Ref	0.75 (0.64–0.86)
Multivariable	Ref	1.08 (0.91–1.28)	0.81 (0.75–0.89)	Ref	0.86 (0.73–1.00)
SERDs					
Univariable	Ref	0.70 (0.47–0.99)	0.93 (0.78–1.11)	Ref	0.95 (0.70–1.26)
Multivariable	Ref	0.71 (0.48–1.03)	0.91 (0.76–1.09)	Ref	1.18 (0.85–1.60)
SERMs					
Univariable	Ref	1.34 (0.99–1.79)	0.54 (0.40–0.72)	Ref	0.75 (0.50–1.10)
Multivariable	Ref	1.37 (1.01–1.85)	0.58 (0.43–0.77)	Ref	0.82 (0.54–1.21)

OSF, ovarian function suppression; AI, aromatase inhibitors; SERDs, selective estrogen receptor degraders; SERM, selective estrogen receptor modulators.

Given the potential influence of age on neuropsychiatric toxicity, we performed a stratified disproportionality analysis based on patient age groups. Stratified analysis revealed distinct profiles of neurologic safety signals across different age cohorts (Supplementary Figures 4A–C). Patients aged >60 years presented the broadest spectrum of neurotoxic events, encompassing common symptoms such as paraesthesia, headache, and peripheral neuropathy, as well as signals related to cerebrovascular events and cognitive impairment (Supplementary Figures 4C). In the 45–60 years group, the number of cerebrovascular signals decreased compared to the older group, while signals unique to this cohort included brain oedema, increased intracranial pressure, and posterior reversible encephalopathy syndrome (Supplementary Figures 4B). Among patients younger than 45 years, fewer neurologic signals were identified overall, which were concentrated on sensory disturbances, peripheral neuropathy, headache, and a limited number of specific neurological syndromes (Supplementary Figures 4A). These findings suggested that older patients may face a more complex and extensive neurotoxicity profile, potentially related to their elevated baseline risk for cerebrovascular diseases and greater comorbidity burden with advancing age. Notably, several signals, including paraesthesia, peripheral neuropathy, and headache, were consistently observed across all age strata, suggesting that these may represent relatively common neurologic events reported with endocrine therapy regardless of age. Psychiatric signals also showed both shared and age-related patterns. Depression, insomnia, and mood changes were common across age groups, whereas younger patients reported relatively stronger signals related to suicidal ideation (Supplementary Figures 5A–C).

### Time to onset and patient outcomes

**FIGURE 8:**
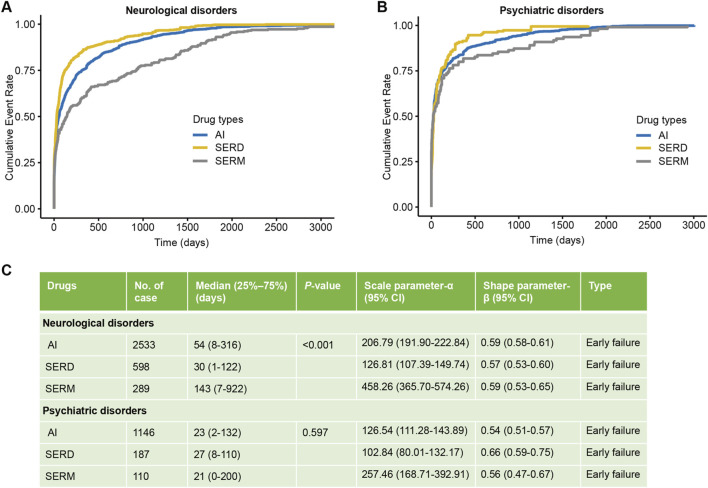
Time-to-onset and pattern analysis of neurologic and psychiatric events across different endocrine therapy classes. **(A)** Cumulative pattern of neurologic events by time-to-onset; **(B)** Cumulative pattern of psychiatric events by time-to-onset; **(C)** Weibull distribution shape analysis of event onset patterns. SERDs, selective estrogen receptor degraders; SERMs, selective estrogen receptor modulators; AIs, aromatase inhibitors.

We then analyzed the time to onset of neuropsychiatric adverse events across different endocrine therapy classes (Figures 8A,B). Neurotoxicity occurred significantly earlier in users of AIs and SERDs compared to SERMs (P < 0.001). The median time to onset was 54 days (interquartile range: 8–316) for AIs and 30 days (interquartile range: 1–122) for SERDs, whereas for SERMs it was 143 days (7–922). Weibull distribution analysis indicated that all three drug classes followed an early failure pattern for neurotoxicity, with β parameters ranging from 0.57 to 0.59, suggesting a decreasing hazard of occurrence over time (Figure 8C). Psychiatric adverse events tended to occur relatively early across all endocrine therapies, typically within the first month of use. The median time to onset was 23 days (range: 2–132) for AIs, 27 days (8–110) for SERDs, and 21 days (0–200) for SERMs. Consistent with neurotoxicity, the Weibull analysis showed a declining trend over time for psychiatric events as well, with β values between 0.54 and 0.66, further supporting the early failure model (Figure 8C). To mitigate potential bias from reporting delays, we performed a sensitivity analysis by excluding cases with a TTO exceeding 1 year. Although median TTO generally shortened because of the exclusion of patients with longer TTO, the pattern of onset remained consistent with the primary analysis. Weibull distribution indicated that both neurological and psychiatric toxicities still exhibited early failure model (Supplementary Figures 6).

A majority of patients experiencing neuropsychiatric adverse events reported serious clinical outcomes, with 60%–80% categorized as severe. Beyond non-specific severity classifications, the most commonly reported outcome was hospitalization or prolonged hospital stay. The distribution of specific types of serious outcomes related to neurotoxicity and psychiatric toxicity across different endocrine therapies is detailed in Supplementary Table 3.

## Discussion

This large-scale pharmacovigilance study is the first to systematically assess the neuropsychiatric safety profiles of the three major classes of endocrine therapies used in breast cancer treatment. Our findings indicate heterogeneous patterns of neuropsychiatric safety signals and reporting burdens across drug classes, offering new insights relevant to clinical monitoring practices. Current real-world data indicate that headache, dizziness, paraesthesia, and peripheral neuropathy are among the most frequently reported neurological adverse events, while insomnia, depression, and anxiety dominate the psychiatric domain. However, notable disproportionality exists across therapies. SERMs demonstrated the relatively strong signals related to cerebrovascular events, including carotid artery dissection, cerebral venous thrombosis, dural arteriovenous fistula, and superior sagittal sinus thrombosis. In contrast, AIs were more frequently associated with peripheral neurotoxic events such as paraesthesia and carpal tunnel syndrome. Moreover, both SERMs and AIs, but not SERDs, showed potential signals of depression-related events. These findings suggest class-specific disproportionality in the reporting of neuropsychiatric events, indicating the importance of individualized monitoring based on the type of endocrine therapy. All three endocrine therapy classes exhibited an “early failure” pattern in time-to-onset analyses for neuropsychiatric events. However, the median time to onset of neurological events was significantly earlier in AI and SERD users compared to SERM users.

Previous evidence on neuropsychiatric toxicity of endocrine therapy in breast cancer has been limited and largely based on small cohorts. Our study identified multiple neuropsychiatric safety signals associated with endocrine therapy for breast cancer at a global pharmacovigilance level. For example, tamoxifen is known to cross the blood-brain barrier and affect dopaminergic pathways; previous animal studies and cognitive research have suggested potential neurological effects (Chen et al., 2002; 2014; Denk et al., 2015; Novick et al., 2020). Here, we identified strong pharmacovigilance signals linking tamoxifen to rare but serious intracranial vascular events. The association between AIs and carpal tunnel syndrome has been observed in retrospective studies and animal models (Chien et al., 2020; Collin et al., 2021; Chung et al., 2022), and our analysis further supports a disproportionate reporting of peripheral sensory abnormalities. Notably, we also observed that letrozole may be associated with spinal cord-related events such as spondylitic myelopathy. For elacestrant, due to its recent market approval, little was previously known about its neuropsychiatric safety profile (Fan et al., 2025; Lv et al., 2025). This study reveals some potential neuropsychiatric events associated with elacestrant based on post-marketing data.

Previous studies have provided some biological evidence supporting the plausibility linking endocrine therapy to central/peripheral neurotoxicity. First, estrogens play critical roles in the brain, regulating neuronal development, synaptic plasticity, excitability, and neuroinflammation (Cersosimo and Benarroch, 2015; Russell et al., 2019; Santoro et al., 2021). These actions are mediated in part through estrogen receptors, which are expressed in dopamine-rich brain regions involved in cognition, affect, and motor function, such as the hippocampus and dorsal striatum (Gogos and van den Buuse, 2015; Almey et al., 2016; Vajaria and Vasudevan, 2018). Consequently, estrogen pathway inhibitors that could penetrate the blood-brain barrier, including aromatase inhibitors and tamoxifen,may disrupt function in these brain regions, potentially leading to cognitive, affective, or motor dysfunction (Curtaz et al., 2022). Second, certain endocrine therapies exhibit pharmacological activities independent of direct interaction with estrogen receptors. For example, tamoxifen has been reported to inhibit protein kinase C signaling and modulate dopamine transport, both of which are important for neuroplasticity, neuronal excitability, and synaptic transmission (Carpenter et al., 2017; Mikelman et al., 2017; Novick et al., 2020). Endocrine therapy may also indirectly increase the risk of neurovascular events by altering coagulation tendency or vascular endothelial responses (Matthews et al., 2018; Blondon et al., 2022). Of note, these biological data offer hypothetical explanations for neuropsychiatric safety signals observed in endocrine therapy, definitive causal mechanisms remain to be elucidated. Beyond the direct or indirect off-target effects of these drugs, alternative contributing factors should also be considered. The underlying tumor burden itself, along with the overall experience of cancer treatment, particularly invasive procedures such as surgery, may affect patients’ neuropsychiatric symptoms (Olson and Marks, 2019; Demos-Davies et al., 2024; Movahed et al., 2025). Furthermore, the clinical course of cancer and patients’ response or outcomes to treatment are closely linked to variations in psychological symptom reporting (Shalata et al., 2024; Yang et al., 2025). Together, these complexities highlight the need for further research to clarify the mechanisms underlying neuropsychiatric events and to explain why different endocrine agents appear to be associated with distinct neuropsychiatric profiles.

The findings of this study suggest the importance of clinician awareness regarding potential neuropsychiatric toxicities of endocrine therapies. Particularly during the early phase of treatment, proactive psychiatric screening and psychological support may be beneficial. Given that the median time to psychiatric event onset was within 1 month for all three classes, early-phase mental health monitoring appears critical. In contrast, neurological events tended to occur later, especially with SERMs, where the median onset time exceeded 3 months. These observations suggest that monitoring strategies could be tailored according to drug class. For example, SERMs may require closer monitoring for intracranial vascular events, especially hemorrhagic events, whereas AIs users may benefit from earlier assessment for peripheral neuropathy and sleep disturbances. For elacestrant, given its shorter post-approval observation window, enhanced pharmacovigilance and targeted research, particularly regarding cognitive impairment, may be warranted.

Despite the strengths of leveraging global pharmacovigilance data, several important limitations must be acknowledged. First, the FAERS and VigiAccess databases rely on spontaneous reporting, which is subject to inherent reporting bias. Adverse event reporting may be influenced by media attention, regulatory actions, and the publication of scientific studies, and mild or expected adverse events are more likely to be underreported than severe or newly recognized events (Hazell and Shakir, 2006; Matsuda et al., 2015). Second, although we standardized all reported events to the preferred term level using MedDRA, variability in adverse event terminology across different reporting sources may still impact data consistency (Garmann et al., 2025). Third, the disproportionality analysis reflects differences in relative reporting frequencies and should not be interpreted as direct comparisons of safety or risk across different drugs. In the absence of total number of patients exposed to each drug, reporting proportions cannot be equated with incidence rates (Fusaroli et al., 2024). Fourth, because of missing or incomplete information, confounding by unmeasured factors, including drug dose, duration of therapy, prior medication history, and preexisting neurological or psychiatric conditions, cannot be excluded. Fifth, as discussed earlier, tumor burden (such as central nervous system involvement) and patients’ response to therapy may independently contribute to neuropsychiatric symptoms (Olson and Marks, 2019; Demos-Davies et al., 2024; Movahed et al., 2025; Shalata et al., 2024; Yang et al., 2025). Although we restricted adverse event reports to those in which endocrine therapy was listed as the primary suspected drug to mitigate the influence of competing factors, residual confounding remains possible. Taken together, these limitations indicate that the findings of this study should be regarded as hypothesis-generating. They reveal safety signals that warrant further investigation but do not yet support causal inference.

## Conclusion

This study provides the first comprehensive pharmacovigilance assessment of the neuropsychiatric burden associated with endocrine therapies in breast cancer. Our findings reveal distinct and clinically relevant safety signal profiles across SERM, SERD, and AI therapies. These results support the rationale for a more personalized approach to patient monitoring and treatment selection, informed by the differential neuropsychiatric toxicity patterns observed across drug classes. Nevertheless, given the limitations of spontaneous reporting data, these associations should be interpreted cautiously as reporting disproportionalities rather than evidence of causality or definitive conclusions. Further confirmation in complementary study designs, including prospective clinical and mechanistic studies, is needed to better characterize and clarify these findings.

## Data Availability

The original contributions presented in the study are included in the article/Supplementary Material, further inquiries can be directed to the corresponding author.
