# Supplementary material for: Profiles of neuropsychiatric toxicity associated with different endocrine therapies for breast cancer: a global pharmacovigilance study based on FAERS and VigiAccess
Source: Front Pharmacol. 2026 Mar 5;17:1731849. doi: 10.3389/fphar.2026.1731849 (PMC12999947; doi:10.3389/fphar.2026.1731849)
Supplement: Supplementary file 1 [file Supplementaryfile1.docx]

| **Supplementary Table 1. Neurologic safety signals for endocrine therapy in breast cancer** | | | | |
| --- | --- | --- | --- | --- |
| **Endocrine Therapeutics** | **Adverse event** | **No. of event** | **ROR (95% CI)** | **EIC (EIC-2SD)** |
| Total drugs | Ageusia | 120 | 1.25 (1.05 - 1.50) | 0.32 (0.06) |
| Total drugs | Amnesia | 324 | 1.27 (1.14 - 1.42) | 0.34 (0.18) |
| Total drugs | Axonal neuropathy | 9 | 2.87 (1.49 - 5.54) | 1.26 (0.34) |
| Total drugs | Basal ganglia infarction | 6 | 4.65 (2.07 - 10.44) | 1.60 (0.49) |
| Total drugs | Brachial plexopathy | 6 | 7.10 (3.15 - 15.98) | 1.90 (0.79) |
| Total drugs | Brain fog | 50 | 1.59 (1.20 - 2.10) | 0.65 (0.24) |
| Total drugs | Burning feet syndrome | 6 | 6.86 (3.04 - 15.44) | 1.88 (0.77) |
| Total drugs | Carotid artery aneurysm | 8 | 6.27 (3.10 - 12.64) | 1.96 (0.99) |
| Total drugs | Carpal tunnel syndrome | 343 | 6.51 (5.85 - 7.25) | 2.65 (2.49) |
| Total drugs | Central nervous system vasculitis | 9 | 2.48 (1.29 - 4.79) | 1.10 (0.19) |
| Total drugs | Cerebral artery embolism | 10 | 2.08 (1.11 - 3.87) | 0.91 (0.04) |
| Total drugs | Cerebral infarction | 132 | 1.39 (1.17 - 1.65) | 0.47 (0.22) |
| Total drugs | Cerebral ischaemia | 47 | 2.27 (1.70 - 3.03) | 1.14 (0.72) |
| Total drugs | Cerebral microangiopathy | 5 | 5.60 (2.31 - 13.61) | 1.65 (0.45) |
| Total drugs | Cerebral microhaemorrhage | 7 | 6.49 (3.06 - 13.76) | 1.92 (0.89) |
| Total drugs | Cerebral thrombosis | 28 | 2.57 (1.77 - 3.73) | 1.28 (0.74) |
| Total drugs | Cerebral venous sinus thrombosis | 15 | 2.10 (1.27 - 3.50) | 0.97 (0.24) |
| Total drugs | Cerebral venous thrombosis | 51 | 7.44 (5.63 - 9.83) | 2.69 (2.29) |
| Total drugs | Complex regional pain syndrome | 12 | 1.97 (1.12 - 3.48) | 0.87 (0.07) |
| Total drugs | Decreased vibratory sense | 7 | 4.94 (2.34 - 10.45) | 1.71 (0.68) |
| Total drugs | Diabetic hyperosmolar coma | 7 | 3.99 (1.89 - 8.42) | 1.52 (0.49) |
| Total drugs | Dural arteriovenous fistula | 12 | 33.39 (18.25 - 61.12) | 3.20 (2.35) |
| Total drugs | Dysaesthesia | 37 | 3.32 (2.40 - 4.59) | 1.63 (1.16) |
| Total drugs | Dysgeusia | 336 | 1.14 (1.02 - 1.27) | 0.19 (0.03) |
| Total drugs | Hashimoto's encephalopathy | 7 | 26.71 (12.22 - 58.35) | 2.63 (1.55) |
| Total drugs | Hemianopia homonymous | 8 | 2.86 (1.43 - 5.75) | 1.24 (0.27) |
| Total drugs | Horner's syndrome | 8 | 3.99 (1.98 - 8.03) | 1.57 (0.60) |
| Total drugs | Hypoaesthesia | 809 | 1.38 (1.29 - 1.48) | 0.46 (0.36) |
| Total drugs | Hypokinesia | 75 | 1.33 (1.06 - 1.67) | 0.41 (0.08) |
| Total drugs | Ischaemic stroke | 175 | 2.56 (2.21 - 2.97) | 1.33 (1.12) |
| Total drugs | Loss of proprioception | 7 | 5.87 (2.77 - 12.43) | 1.85 (0.81) |
| Total drugs | Meningeal disorder | 14 | 8.95 (5.25 - 15.26) | 2.52 (1.76) |
| Total drugs | Migraine with aura | 20 | 1.95 (1.26 - 3.03) | 0.90 (0.26) |
| Total drugs | Monoplegia | 45 | 2.68 (2.00 - 3.59) | 1.36 (0.93) |
| Total drugs | Movement disorder | 184 | 1.48 (1.28 - 1.71) | 0.56 (0.35) |
| Total drugs | Myelomalacia | 4 | 10.28 (3.78 - 27.98) | 1.83 (0.50) |
| Total drugs | Neuralgia | 158 | 1.73 (1.48 - 2.02) | 0.78 (0.55) |
| Total drugs | Neuropathy peripheral | 792 | 2.25 (2.10 - 2.41) | 1.16 (1.05) |
| Total drugs | Ophthalmic migraine | 6 | 3.33 (1.49 - 7.46) | 1.31 (0.21) |
| Total drugs | Paraesthesia | 783 | 1.28 (1.19 - 1.37) | 0.35 (0.25) |
| Total drugs | Paralysis recurrent laryngeal nerve | 5 | 10.56 (4.31 - 25.87) | 2.00 (0.80) |
| Total drugs | Paraparesis | 16 | 2.31 (1.41 - 3.78) | 1.09 (0.39) |
| Total drugs | Paresis | 20 | 2.26 (1.46 - 3.52) | 1.09 (0.45) |
| Total drugs | Parosmia | 48 | 1.69 (1.27 - 2.24) | 0.73 (0.31) |
| Total drugs | Periodic limb movement disorder | 6 | 4.24 (1.89 - 9.50) | 1.52 (0.42) |
| Total drugs | Peripheral nerve lesion | 12 | 8.76 (4.92 - 15.59) | 2.43 (1.61) |
| Total drugs | Peripheral sensory neuropathy | 56 | 2.61 (2.01 - 3.40) | 1.33 (0.95) |
| Total drugs | Polyneuropathy | 271 | 6.25 (5.54 - 7.06) | 2.58 (2.41) |
| Total drugs | Psychomotor disadaptation syndrome | 3 | 20.27 (6.24 - 65.92) | 1.78 (0.27) |
| Total drugs | Radicular pain | 9 | 6.63 (3.42 - 12.86) | 2.06 (1.14) |
| Total drugs | Radiculopathy | 34 | 2.43 (1.73 - 3.40) | 1.21 (0.72) |
| Total drugs | Reversible cerebral vasoconstriction syndrome | 24 | 3.66 (2.45 - 5.48) | 1.71 (1.13) |
| Total drugs | Sciatica | 124 | 2.13 (1.78 - 2.54) | 1.07 (0.81) |
| Total drugs | Sensory disturbance | 83 | 1.30 (1.05 - 1.62) | 0.38 (0.06) |
| Total drugs | Spinal cord compression | 33 | 2.17 (1.54 - 3.06) | 1.06 (0.57) |
| Total drugs | Spinal cord haematoma | 12 | 9.75 (5.48 - 17.38) | 2.51 (1.69) |
| Total drugs | Spinal cord oedema | 5 | 3.57 (1.48 - 8.64) | 1.31 (0.12) |
| Total drugs | Spondylitic myelopathy | 4 | 14.34 (5.22 - 39.33) | 1.94 (0.61) |
| Total drugs | Superior sagittal sinus thrombosis | 9 | 2.80 (1.45 - 5.40) | 1.24 (0.32) |
| Total drugs | Taste disorder | 136 | 2.05 (1.74 - 2.43) | 1.02 (0.77) |
| Total drugs | Thrombotic cerebral infarction | 5 | 3.44 (1.42 - 8.31) | 1.28 (0.09) |
| Total drugs | Toxic neuropathy | 5 | 3.29 (1.36 - 7.96) | 1.24 (0.05) |
| Total drugs | Vocal cord paralysis | 19 | 2.76 (1.75 - 4.33) | 1.33 (0.68) |
| Total drugs | White matter lesion | 11 | 2.31 (1.28 - 4.18) | 1.05 (0.21) |
| Tamoxifen | Amnesia | 28 | 1.66 (1.15 - 2.41) | 0.70 (0.16) |
| Tamoxifen | Amnestic disorder | 4 | 32.78 (12.25 - 87.73) | 2.15 (0.86) |
| Tamoxifen | Anosmia | 9 | 2.46 (1.28 - 4.72) | 1.10 (0.19) |
| Tamoxifen | Atonic seizures | 3 | 17.58 (5.65 - 54.65) | 1.77 (0.32) |
| Tamoxifen | Carotid artery dissection | 4 | 36.80 (13.74 - 98.56) | 2.17 (0.87) |
| Tamoxifen | Carotid artery occlusion | 4 | 4.25 (1.59 - 11.33) | 1.36 (0.07) |
| Tamoxifen | Carpal tunnel syndrome | 8 | 2.25 (1.12 - 4.50) | 0.98 (0.02) |
| Tamoxifen | Cerebellar haemorrhage | 4 | 10.63 (3.98 - 28.36) | 1.86 (0.57) |
| Tamoxifen | Cerebellar infarction | 3 | 6.90 (2.22 - 21.42) | 1.48 (0.03) |
| Tamoxifen | Cerebral haemorrhage | 20 | 2.20 (1.42 - 3.41) | 1.06 (0.43) |
| Tamoxifen | Cerebral infarction | 28 | 4.47 (3.08 - 6.47) | 1.99 (1.46) |
| Tamoxifen | Cerebral ischaemia | 6 | 4.37 (1.96 - 9.74) | 1.56 (0.47) |
| Tamoxifen | Cerebral venous sinus thrombosis | 4 | 8.47 (3.18 - 22.61) | 1.76 (0.47) |
| Tamoxifen | Cerebral venous thrombosis | 26 | 56.87 (38.59 - 83.80) | 4.20 (3.64) |
| Tamoxifen | Cerebrovascular accident | 61 | 1.39 (1.08 - 1.79) | 0.47 (0.10) |
| Tamoxifen | Cognitive disorder | 23 | 1.97 (1.31 - 2.97) | 0.92 (0.33) |
| Tamoxifen | Disturbance in attention | 40 | 2.86 (2.10 - 3.90) | 1.45 (1.00) |
| Tamoxifen | Dural arteriovenous fistula | 11 | 460.33 (245.70 - 862.44) | 3.55 (2.66) |
| Tamoxifen | Hemiplegia | 8 | 3.71 (1.86 - 7.43) | 1.51 (0.55) |
| Tamoxifen | Horner's syndrome | 4 | 30.10 (11.25 - 80.55) | 2.14 (0.84) |
| Tamoxifen | Intracranial pressure increased | 5 | 3.57 (1.49 - 8.58) | 1.32 (0.14) |
| Tamoxifen | Ischaemic stroke | 22 | 4.85 (3.19 - 7.38) | 2.05 (1.45) |
| Tamoxifen | Meningeal disorder | 3 | 28.32 (9.09 - 88.22) | 1.85 (0.40) |
| Tamoxifen | Migraine with aura | 5 | 7.38 (3.07 - 17.74) | 1.84 (0.66) |
| Tamoxifen | Monoplegia | 8 | 7.18 (3.59 - 14.37) | 2.09 (1.12) |
| Tamoxifen | Neuropathy peripheral | 53 | 2.27 (1.73 - 2.97) | 1.14 (0.75) |
| Tamoxifen | Optic neuritis | 13 | 5.51 (3.20 - 9.50) | 2.06 (1.29) |
| Tamoxifen | Pachymeningitis | 3 | 47.96 (15.35 - 149.85) | 1.91 (0.46) |
| Tamoxifen | Sensory loss | 9 | 4.40 (2.29 - 8.45) | 1.71 (0.80) |
| Tamoxifen | Small fibre neuropathy | 3 | 18.83 (6.06 - 58.57) | 1.79 (0.34) |
| Tamoxifen | Superior sagittal sinus thrombosis | 7 | 33.05 (15.70 - 69.58) | 2.72 (1.69) |
| Tamoxifen | Transient ischaemic attack | 26 | 2.97 (2.02 - 4.36) | 1.47 (0.91) |
| Tamoxifen | Transverse sinus thrombosis | 4 | 29.13 (10.89 - 77.93) | 2.13 (0.84) |
| Toremifene | Subarachnoid haemorrhage | 4 | 31.47 (11.78 - 84.08) | 2.15 (0.85) |
| Fulvestrant | Brain oedema | 23 | 2.83 (1.88 - 4.26) | 1.39 (0.80) |
| Fulvestrant | Cerebral venous thrombosis | 6 | 5.03 (2.26 - 11.22) | 1.67 (0.58) |
| Fulvestrant | Complex regional pain syndrome | 4 | 3.86 (1.45 - 10.30) | 1.29 (0.00) |
| Fulvestrant | Hypoaesthesia | 140 | 1.41 (1.19 - 1.66) | 0.49 (0.24) |
| Fulvestrant | Intracranial pressure increased | 9 | 2.50 (1.30 - 4.81) | 1.12 (0.21) |
| Fulvestrant | Movement disorder | 41 | 1.94 (1.43 - 2.64) | 0.92 (0.48) |
| Fulvestrant | Neuralgia | 35 | 2.25 (1.61 - 3.13) | 1.12 (0.64) |
| Fulvestrant | Neuropathy peripheral | 109 | 1.81 (1.50 - 2.19) | 0.84 (0.57) |
| Fulvestrant | Parosmia | 19 | 3.93 (2.50 - 6.16) | 1.77 (1.13) |
| Fulvestrant | Peroneal nerve palsy | 9 | 2.40 (1.25 - 4.62) | 1.07 (0.16) |
| Fulvestrant | Polyneuropathy | 59 | 7.89 (6.10 - 10.19) | 2.81 (2.44) |
| Fulvestrant | Posterior reversible encephalopathy syndrome | 15 | 2.32 (1.40 - 3.84) | 1.10 (0.37) |
| Fulvestrant | Radicular pain | 3 | 12.83 (4.11 - 39.98) | 1.69 (0.24) |
| Fulvestrant | Reversible cerebral vasoconstriction syndrome | 6 | 5.35 (2.40 - 11.93) | 1.72 (0.63) |
| Fulvestrant | Sciatic nerve neuropathy | 3 | 8.56 (2.75 - 26.63) | 1.56 (0.12) |
| Fulvestrant | Sciatica | 48 | 4.85 (3.65 - 6.43) | 2.16 (1.75) |
| Fulvestrant | Sensory disturbance | 36 | 3.34 (2.41 - 4.63) | 1.65 (1.17) |
| Fulvestrant | Spinal cord compression | 7 | 2.71 (1.29 - 5.68) | 1.16 (0.13) |
| Fulvestrant | Spinal cord haematoma | 12 | 57.66 (32.37 - 102.72) | 3.42 (2.60) |
| Fulvestrant | Taste disorder | 40 | 3.55 (2.60 - 4.84) | 1.74 (1.29) |
| Fulvestrant | Vocal cord paralysis | 12 | 10.25 (5.81 - 18.09) | 2.58 (1.77) |
| Fulvestrant | White matter lesion | 6 | 7.42 (3.32 - 16.55) | 1.95 (0.85) |
| Elacestrant | Ageusia | 15 | 2.08 (1.26 - 3.46) | 0.96 (0.24) |
| Elacestrant | Brain fog | 17 | 7.19 (4.46 - 11.57) | 2.42 (1.73) |
| Elacestrant | Hypersomnia | 17 | 2.08 (1.29 - 3.34) | 0.97 (0.29) |
| Elacestrant | Lethargy | 26 | 1.56 (1.06 - 2.29) | 0.61 (0.05) |
| Elacestrant | Neuralgia | 14 | 2.03 (1.20 - 3.43) | 0.92 (0.18) |
| Elacestrant | Neuropathy peripheral | 42 | 1.57 (1.16 - 2.13) | 0.63 (0.19) |
| Elacestrant | Sciatica | 11 | 2.50 (1.38 - 4.51) | 1.15 (0.32) |
| Elacestrant | Taste disorder | 20 | 4.00 (2.58 - 6.21) | 1.81 (1.18) |
| Anastrozole | Amnesia | 134 | 2.19 (1.85 - 2.60) | 1.12 (0.87) |
| Anastrozole | Brain fog | 14 | 1.86 (1.10 - 3.13) | 0.81 (0.06) |
| Anastrozole | Burning sensation | 111 | 1.72 (1.43 - 2.07) | 0.77 (0.50) |
| Anastrozole | Carpal tunnel syndrome | 184 | 14.47 (12.51 - 16.74) | 3.73 (3.52) |
| Anastrozole | Central nervous system vasculitis | 8 | 9.23 (4.60 - 18.53) | 2.26 (1.30) |
| Anastrozole | Cerebral ischaemia | 14 | 2.81 (1.66 - 4.75) | 1.32 (0.58) |
| Anastrozole | Cerebral microhaemorrhage | 5 | 19.27 (7.95 - 46.69) | 2.25 (1.05) |
| Anastrozole | Cerebral thrombosis | 10 | 3.81 (2.05 - 7.10) | 1.60 (0.73) |
| Anastrozole | Cognitive disorder | 61 | 1.44 (1.12 - 1.85) | 0.52 (0.15) |
| Anastrozole | Cubital tunnel syndrome | 3 | 9.55 (3.06 - 29.78) | 1.60 (0.15) |
| Anastrozole | Dementia alzheimer's type | 28 | 3.28 (2.26 - 4.75) | 1.60 (1.06) |
| Anastrozole | Diabetic hyperosmolar coma | 6 | 14.28 (6.38 - 31.98) | 2.29 (1.19) |
| Anastrozole | Diplegia | 7 | 3.78 (1.80 - 7.94) | 1.48 (0.46) |
| Anastrozole | Dysgeusia | 102 | 1.45 (1.19 - 1.76) | 0.52 (0.24) |
| Anastrozole | Dysstasia | 50 | 1.84 (1.39 - 2.42) | 0.85 (0.45) |
| Anastrozole | Haemorrhagic stroke | 15 | 2.03 (1.22 - 3.36) | 0.93 (0.20) |
| Anastrozole | Haemorrhagic transformation stroke | 5 | 3.59 (1.49 - 8.64) | 1.32 (0.14) |
| Anastrozole | Head discomfort | 29 | 1.85 (1.29 - 2.67) | 0.85 (0.32) |
| Anastrozole | Headache | 670 | 1.17 (1.08 - 1.26) | 0.22 (0.11) |
| Anastrozole | Horner's syndrome | 4 | 8.29 (3.10 - 22.17) | 1.75 (0.45) |
| Anastrozole | Hypoaesthesia | 292 | 2.08 (1.86 - 2.34) | 1.05 (0.88) |
| Anastrozole | Ischaemic stroke | 44 | 2.67 (1.99 - 3.60) | 1.36 (0.93) |
| Anastrozole | Memory impairment | 193 | 1.52 (1.32 - 1.75) | 0.60 (0.39) |
| Anastrozole | Movement disorder | 47 | 1.58 (1.18 - 2.10) | 0.64 (0.22) |
| Anastrozole | Neuralgia | 50 | 2.28 (1.73 - 3.01) | 1.15 (0.74) |
| Anastrozole | Neuropathy peripheral | 235 | 2.78 (2.44 - 3.16) | 1.46 (1.27) |
| Anastrozole | Optic neuritis | 16 | 1.87 (1.14 - 3.05) | 0.83 (0.13) |
| Anastrozole | Paraesthesia | 260 | 1.77 (1.57 - 2.00) | 0.82 (0.64) |
| Anastrozole | Paraparesis | 6 | 3.60 (1.62 - 8.03) | 1.39 (0.30) |
| Anastrozole | Paresis | 8 | 3.77 (1.88 - 7.55) | 1.52 (0.56) |
| Anastrozole | Parosmia | 16 | 2.34 (1.43 - 3.82) | 1.12 (0.41) |
| Anastrozole | Peripheral sensory neuropathy | 11 | 2.13 (1.18 - 3.84) | 0.96 (0.12) |
| Anastrozole | Polyneuropathy | 44 | 4.16 (3.09 - 5.59) | 1.95 (1.52) |
| Anastrozole | Radiculopathy | 8 | 2.37 (1.19 - 4.75) | 1.04 (0.08) |
| Anastrozole | Reversible cerebral vasoconstriction syndrome | 12 | 7.61 (4.31 - 13.43) | 2.33 (1.52) |
| Anastrozole | Sciatica | 30 | 2.14 (1.50 - 3.06) | 1.04 (0.53) |
| Anastrozole | Tension headache | 10 | 2.56 (1.38 - 4.77) | 1.16 (0.29) |
| Anastrozole | Tongue biting | 8 | 4.22 (2.11 - 8.45) | 1.63 (0.67) |
| Anastrozole | Transient ischaemic attack | 46 | 1.45 (1.08 - 1.93) | 0.52 (0.10) |
| Letrozole | Axonal neuropathy | 8 | 7.34 (3.66 - 14.74) | 2.10 (1.13) |
| Letrozole | Basal ganglia infarction | 6 | 13.42 (5.98 - 30.10) | 2.26 (1.16) |
| Letrozole | Brachial plexopathy | 6 | 20.46 (9.08 - 46.09) | 2.43 (1.32) |
| Letrozole | Carotid artery aneurysm | 5 | 11.18 (4.62 - 27.05) | 2.04 (0.85) |
| Letrozole | Carotid artery occlusion | 10 | 2.02 (1.08 - 3.75) | 0.88 (0.01) |
| Letrozole | Carpal tunnel syndrome | 90 | 4.83 (3.92 - 5.94) | 2.20 (1.90) |
| Letrozole | Cerebral artery embolism | 6 | 3.58 (1.60 - 7.98) | 1.38 (0.29) |
| Letrozole | Cerebral haematoma | 11 | 2.43 (1.34 - 4.39) | 1.11 (0.28) |
| Letrozole | Cerebral infarction | 47 | 1.42 (1.07 - 1.89) | 0.49 (0.08) |
| Letrozole | Cerebral ischaemia | 15 | 2.08 (1.25 - 3.45) | 0.96 (0.23) |
| Letrozole | Cerebral microangiopathy | 4 | 12.87 (4.78 - 34.60) | 1.92 (0.62) |
| Letrozole | Cerebral thrombosis | 11 | 2.89 (1.60 - 5.22) | 1.32 (0.48) |
| Letrozole | Cerebral venous thrombosis | 14 | 5.76 (3.40 - 9.74) | 2.12 (1.37) |
| Letrozole | Cervicobrachial syndrome | 4 | 4.48 (1.68 - 11.97) | 1.40 (0.10) |
| Letrozole | Coordination abnormal | 30 | 1.56 (1.09 - 2.23) | 0.61 (0.09) |
| Letrozole | Decreased vibratory sense | 6 | 12.18 (5.43 - 27.30) | 2.22 (1.12) |
| Letrozole | Dementia | 48 | 1.35 (1.02 - 1.79) | 0.42 (0.01) |
| Letrozole | Dysaesthesia | 22 | 5.66 (3.72 - 8.61) | 2.23 (1.62) |
| Letrozole | Dyslalia | 4 | 4.02 (1.51 - 10.75) | 1.32 (0.03) |
| Letrozole | Hashimoto's encephalopathy | 7 | 76.99 (35.24 - 168.22) | 2.86 (1.78) |
| Letrozole | Hemianopia homonymous | 6 | 6.17 (2.76 - 13.79) | 1.82 (0.72) |
| Letrozole | Hypokinesia | 34 | 1.74 (1.24 - 2.44) | 0.77 (0.28) |
| Letrozole | Ischaemic stroke | 80 | 3.36 (2.69 - 4.18) | 1.70 (1.38) |
| Letrozole | Loss of proprioception | 6 | 14.46 (6.44 - 32.46) | 2.30 (1.19) |
| Letrozole | Meningeal disorder | 7 | 12.66 (5.99 - 26.74) | 2.35 (1.32) |
| Letrozole | Migraine with aura | 11 | 3.08 (1.71 - 5.58) | 1.39 (0.55) |
| Letrozole | Monoplegia | 27 | 4.61 (3.16 - 6.74) | 2.02 (1.48) |
| Letrozole | Movement disorder | 74 | 1.71 (1.36 - 2.15) | 0.76 (0.42) |
| Letrozole | Myelomalacia | 4 | 29.65 (10.90 - 80.68) | 2.13 (0.81) |
| Letrozole | Neuropathy peripheral | 275 | 2.24 (1.99 - 2.52) | 1.15 (0.97) |
| Letrozole | Ophthalmic migraine | 6 | 9.60 (4.29 - 21.50) | 2.10 (1.00) |
| Letrozole | Paraesthesia | 251 | 1.18 (1.04 - 1.33) | 0.23 (0.05) |
| Letrozole | Paralysis recurrent laryngeal nerve | 4 | 24.14 (8.90 - 65.43) | 2.09 (0.77) |
| Letrozole | Paraparesis | 7 | 2.89 (1.38 - 6.08) | 1.22 (0.20) |
| Letrozole | Paresis | 8 | 2.60 (1.30 - 5.20) | 1.14 (0.17) |
| Letrozole | Periodic limb movement disorder | 6 | 12.21 (5.45 - 27.38) | 2.22 (1.12) |
| Letrozole | Peripheral nerve lesion | 7 | 14.51 (6.86 - 30.68) | 2.42 (1.39) |
| Letrozole | Peripheral sensorimotor neuropathy | 8 | 5.27 (2.63 - 10.56) | 1.83 (0.86) |
| Letrozole | Peripheral sensory neuropathy | 29 | 3.88 (2.69 - 5.58) | 1.82 (1.29) |
| Letrozole | Polyneuropathy | 138 | 9.07 (7.67 - 10.73) | 3.08 (2.83) |
| Letrozole | Psychomotor disadaptation syndrome | 3 | 58.45 (17.98 - 190.05) | 1.92 (0.40) |
| Letrozole | Radicular pain | 4 | 8.37 (3.12 - 22.43) | 1.75 (0.45) |
| Letrozole | Radiculopathy | 14 | 2.86 (1.69 - 4.84) | 1.35 (0.60) |
| Letrozole | Sciatica | 32 | 1.57 (1.11 - 2.22) | 0.63 (0.12) |
| Letrozole | Spinal cord compression | 18 | 3.40 (2.14 - 5.41) | 1.59 (0.93) |
| Letrozole | Spondylitic myelopathy | 4 | 41.33 (15.06 - 113.39) | 2.18 (0.84) |
| Letrozole | Sudden onset of sleep | 6 | 3.14 (1.41 - 7.00) | 1.26 (0.17) |
| Letrozole | Taste disorder | 58 | 2.51 (1.94 - 3.25) | 1.29 (0.91) |
| Letrozole | Toxic neuropathy | 4 | 7.58 (2.83 - 20.30) | 1.70 (0.40) |
| Letrozole | Trigeminal nerve disorder | 4 | 8.63 (3.22 - 23.14) | 1.77 (0.47) |
| Exemestane | Amnesia | 37 | 1.48 (1.07 - 2.04) | 0.54 (0.07) |
| Exemestane | Balance disorder | 47 | 1.43 (1.07 - 1.90) | 0.50 (0.08) |
| Exemestane | Carpal tunnel syndrome | 46 | 8.73 (6.53 - 11.66) | 2.90 (2.48) |
| Exemestane | Cerebral infarction | 16 | 1.71 (1.05 - 2.80) | 0.72 (0.02) |
| Exemestane | Cerebral ischaemia | 8 | 3.92 (1.96 - 7.84) | 1.56 (0.60) |
| Exemestane | Cerebral venous thrombosis | 4 | 5.79 (2.17 - 15.46) | 1.56 (0.27) |
| Exemestane | Cerebrovascular accident | 101 | 1.55 (1.28 - 1.89) | 0.62 (0.34) |
| Exemestane | Cognitive disorder | 34 | 1.96 (1.40 - 2.74) | 0.93 (0.44) |
| Exemestane | Dementia | 18 | 1.80 (1.13 - 2.85) | 0.78 (0.12) |
| Exemestane | Dysgeusia | 41 | 1.42 (1.04 - 1.92) | 0.49 (0.04) |
| Exemestane | Hypoaesthesia | 83 | 1.44 (1.16 - 1.79) | 0.52 (0.20) |
| Exemestane | Ischaemic stroke | 19 | 2.82 (1.79 - 4.42) | 1.37 (0.72) |
| Exemestane | Lacunar infarction | 4 | 4.23 (1.58 - 11.27) | 1.36 (0.07) |
| Exemestane | Memory impairment | 90 | 1.73 (1.41 - 2.13) | 0.78 (0.47) |
| Exemestane | Neuralgia | 17 | 1.89 (1.17 - 3.04) | 0.85 (0.16) |
| Exemestane | Neuropathy peripheral | 77 | 2.21 (1.77 - 2.77) | 1.12 (0.79) |
| Exemestane | Paraesthesia | 102 | 1.70 (1.40 - 2.06) | 0.75 (0.46) |
| Exemestane | Peripheral sensory neuropathy | 8 | 3.77 (1.89 - 7.55) | 1.53 (0.56) |
| Exemestane | Polyneuropathy | 26 | 5.99 (4.07 - 8.80) | 2.33 (1.78) |
| Exemestane | Radiculopathy | 5 | 3.62 (1.50 - 8.69) | 1.33 (0.15) |
| Exemestane | Tension headache | 5 | 3.13 (1.30 - 7.52) | 1.21 (0.03) |
| Exemestane | Thrombotic cerebral infarction | 3 | 20.96 (6.73 - 65.31) | 1.80 (0.35) |
| Exemestane | Transient ischaemic attack | 23 | 1.76 (1.17 - 2.65) | 0.77 (0.18) |
| ROR, reporting odds ratio; EIC, the expectation of information component; SD, standard deviation | | | | |

| **Supplementary Table 2. Psychiatric safety signals for endocrine therapy in breast cancer** | | | | |
| --- | --- | --- | --- | --- |
| **Endocrine Therapeutics** | **Adverse event** | **No. of event** | **ROR (95% CI)** | **EIC (EIC-2SD)** |
| Total drugs | Affective disorder | 41 | 1.39 (1.02 - 1.89) | 0.46 (0.01) |
| Total drugs | Burnout syndrome | 7 | 3.27 (1.55 - 6.90) | 1.34 (0.31) |
| Total drugs | Depressed mood | 308 | 1.57 (1.40 - 1.75) | 0.64 (0.48) |
| Total drugs | Depression | 1167 | 1.31 (1.23 - 1.39) | 0.38 (0.30) |
| Total drugs | Depression suicidal | 32 | 2.39 (1.69 - 3.39) | 1.19 (0.68) |
| Total drugs | Discouragement | 15 | 1.85 (1.11 - 3.07) | 0.81 (0.08) |
| Total drugs | Disturbance in sexual arousal | 10 | 2.86 (1.53 - 5.33) | 1.28 (0.40) |
| Total drugs | Eating disorder | 129 | 1.57 (1.32 - 1.86) | 0.64 (0.38) |
| Total drugs | Insomnia | 1464 | 1.43 (1.35 - 1.50) | 0.51 (0.43) |
| Total drugs | Libido decreased | 88 | 1.84 (1.49 - 2.27) | 0.86 (0.56) |
| Total drugs | Libido disorder | 19 | 3.82 (2.43 - 6.01) | 1.73 (1.08) |
| Total drugs | Mixed anxiety and depressive disorder | 9 | 8.62 (4.43 - 16.76) | 2.26 (1.33) |
| Total drugs | Mood altered | 191 | 1.86 (1.61 - 2.14) | 0.88 (0.67) |
| Total drugs | Mood swings | 215 | 1.74 (1.52 - 1.99) | 0.79 (0.59) |
| Total drugs | Sleep disorder | 449 | 1.74 (1.58 - 1.90) | 0.79 (0.65) |
| Tamoxifen | Affective disorder | 7 | 3.59 (1.71 - 7.54) | 1.44 (0.42) |
| Tamoxifen | Depressed mood | 28 | 2.15 (1.49 - 3.12) | 1.05 (0.51) |
| Tamoxifen | Depression | 107 | 1.82 (1.50 - 2.20) | 0.85 (0.57) |
| Tamoxifen | Depression suicidal | 4 | 4.50 (1.69 - 12.01) | 1.40 (0.11) |
| Tamoxifen | Feelings of worthlessness | 3 | 8.53 (2.75 - 26.49) | 1.56 (0.12) |
| Tamoxifen | Hypomania | 4 | 4.25 (1.59 - 11.33) | 1.36 (0.07) |
| Tamoxifen | Insomnia | 107 | 1.58 (1.30 - 1.91) | 0.65 (0.37) |
| Tamoxifen | Irritability | 25 | 1.62 (1.09 - 2.40) | 0.66 (0.09) |
| Tamoxifen | Libido decreased | 11 | 3.48 (1.93 - 6.29) | 1.53 (0.69) |
| Tamoxifen | Loss of libido | 10 | 4.52 (2.43 - 8.40) | 1.77 (0.90) |
| Tamoxifen | Major depression | 13 | 6.96 (4.04 - 11.99) | 2.28 (1.51) |
| Tamoxifen | Mood altered | 17 | 2.49 (1.55 - 4.01) | 1.20 (0.52) |
| Tamoxifen | Phonophobia | 3 | 27.11 (8.71 - 84.44) | 1.85 (0.40) |
| Tamoxifen | Sleep disorder | 27 | 1.58 (1.08 - 2.30) | 0.63 (0.08) |
| Tamoxifen | Suicidal ideation | 57 | 2.46 (1.89 - 3.19) | 1.26 (0.88) |
| Fulvestrant | Acute stress disorder | 4 | 18.58 (6.93 - 49.83) | 2.04 (0.74) |
| Fulvestrant | Eating disorder | 30 | 2.14 (1.50 - 3.07) | 1.05 (0.53) |
| Fulvestrant | Sleep disorder | 73 | 1.66 (1.32 - 2.09) | 0.71 (0.38) |
| Elacestrant | Sleep disorder | 29 | 1.48 (1.03 - 2.14) | 0.55 (0.02) |
| Anastrozole | Depersonalisation/derealisation disorder | 7 | 3.27 (1.56 - 6.88) | 1.35 (0.33) |
| Anastrozole | Depressed mood | 89 | 1.89 (1.53 - 2.32) | 0.90 (0.59) |
| Anastrozole | Depression | 459 | 2.15 (1.96 - 2.36) | 1.09 (0.96) |
| Anastrozole | Depression suicidal | 11 | 3.42 (1.89 - 6.18) | 1.51 (0.67) |
| Anastrozole | Disturbance in sexual arousal | 9 | 10.75 (5.57 - 20.74) | 2.44 (1.52) |
| Anastrozole | Insomnia | 586 | 2.39 (2.20 - 2.59) | 1.24 (1.12) |
| Anastrozole | Irritability | 76 | 1.36 (1.08 - 1.70) | 0.43 (0.10) |
| Anastrozole | Libido decreased | 48 | 4.20 (3.16 - 5.57) | 1.97 (1.56) |
| Anastrozole | Loss of libido | 17 | 2.12 (1.31 - 3.40) | 0.99 (0.31) |
| Anastrozole | Mood altered | 106 | 4.30 (3.55 - 5.21) | 2.05 (1.77) |
| Anastrozole | Mood swings | 118 | 3.98 (3.32 - 4.77) | 1.95 (1.69) |
| Anastrozole | Sleep disorder | 94 | 1.51 (1.24 - 1.85) | 0.59 (0.29) |
| Anastrozole | Sleep disorder due to general medical condition, insomnia type | 11 | 2.10 (1.16 - 3.79) | 0.94 (0.11) |
| Anastrozole | Tearfulness | 16 | 3.96 (2.42 - 6.47) | 1.75 (1.05) |
| Letrozole | Burnout syndrome | 7 | 9.43 (4.47 - 19.89) | 2.19 (1.16) |
| Letrozole | Depressed mood | 124 | 1.81 (1.52 - 2.16) | 0.85 (0.59) |
| Letrozole | Depression suicidal | 14 | 3.00 (1.77 - 5.07) | 1.40 (0.65) |
| Letrozole | Eating disorder | 47 | 1.64 (1.23 - 2.18) | 0.69 (0.27) |
| Letrozole | Libido disorder | 17 | 9.84 (6.10 - 15.89) | 2.71 (2.02) |
| Letrozole | Listless | 11 | 2.49 (1.38 - 4.49) | 1.14 (0.31) |
| Letrozole | Mixed anxiety and depressive disorder | 9 | 24.85 (12.78 - 48.33) | 2.86 (1.93) |
| Letrozole | Obsessive-compulsive symptom | 3 | 6.64 (2.13 - 20.71) | 1.46 (0.01) |
| Letrozole | Phobia of driving | 3 | 14.72 (4.69 - 46.19) | 1.73 (0.26) |
| Letrozole | Sleep disorder | 173 | 1.92 (1.65 - 2.23) | 0.93 (0.71) |
| Letrozole | Substance-induced psychotic disorder | 8 | 2.97 (1.48 - 5.95) | 1.28 (0.32) |
| Letrozole | Tension | 16 | 1.91 (1.17 - 3.12) | 0.86 (0.16) |
| Exemestane | Depression | 144 | 1.64 (1.39 - 1.94) | 0.71 (0.47) |
| Exemestane | Eating disorder | 17 | 2.10 (1.30 - 3.38) | 0.98 (0.30) |
| Exemestane | Insomnia | 232 | 2.31 (2.03 - 2.62) | 1.19 (1.00) |
| Exemestane | Mood swings | 23 | 1.89 (1.25 - 2.84) | 0.86 (0.27) |
| Exemestane | Sleep disorder | 52 | 2.04 (1.56 - 2.68) | 1.00 (0.60) |
| ROR, reporting odds ratio; EIC, the expectation of information component; SD, standard deviation | | | | |

**Supplementary Table 3.** Outcomes of neurological and psychiatric disorders among different endocrine therapies.

| **Groups** | **Outcome** | | | | | | |  |
| --- | --- | --- | --- | --- | --- | --- | --- | --- |
|  | **Total** | **CA** | **DE** | **DS** | **HO** | **LT** | **RI** | **OT** |
| **Neurological disorders** | | | | | | | | |
| AIs | 77.30% | 0.05% | 4.62% | 5.93% | 24.96% | 3.59% | 0.61% | 59.55% |
| SERDs | 63.25% | 0% | 5.77% | 2.69% | 25.04% | 3.42% | 0.47% | 49.83% |
| SERMs | 83.06% | 0.23% | 5.02% | 10.51% | 28.86% | 8.29% | 4.67% | 51.64% |
| **Psychiatric disorders** | | | | | | | | |
| AIs | 69.96% | 0.07% | 3.32% | 6.24% | 17.45% | 3.06% | 0.54% | 57.33% |
| SERDs | 62.65% | 0% | 6.73% | 1.86% | 27.15% | 2.67% | 0.23% | 51.74% |
| SERMs | 79.68% | 0.46% | 3.88% | 9.59% | 17.35% | 8.22% | 3.65% | 58.45% |

CA, congenital anomaly; DE, death; DS, disability; HO, hospitalization - initial or prolonged; LT, life-threatening; RI, required intervention to prevent permanent impairment/damage; OT, other serious outcomes; AI, aromatase inhibitors; SERDs, selective estrogen receptor degraders; SERM, selective estrogen receptor modulators.

Note: multiple serious outcomes may exist in a single patient.


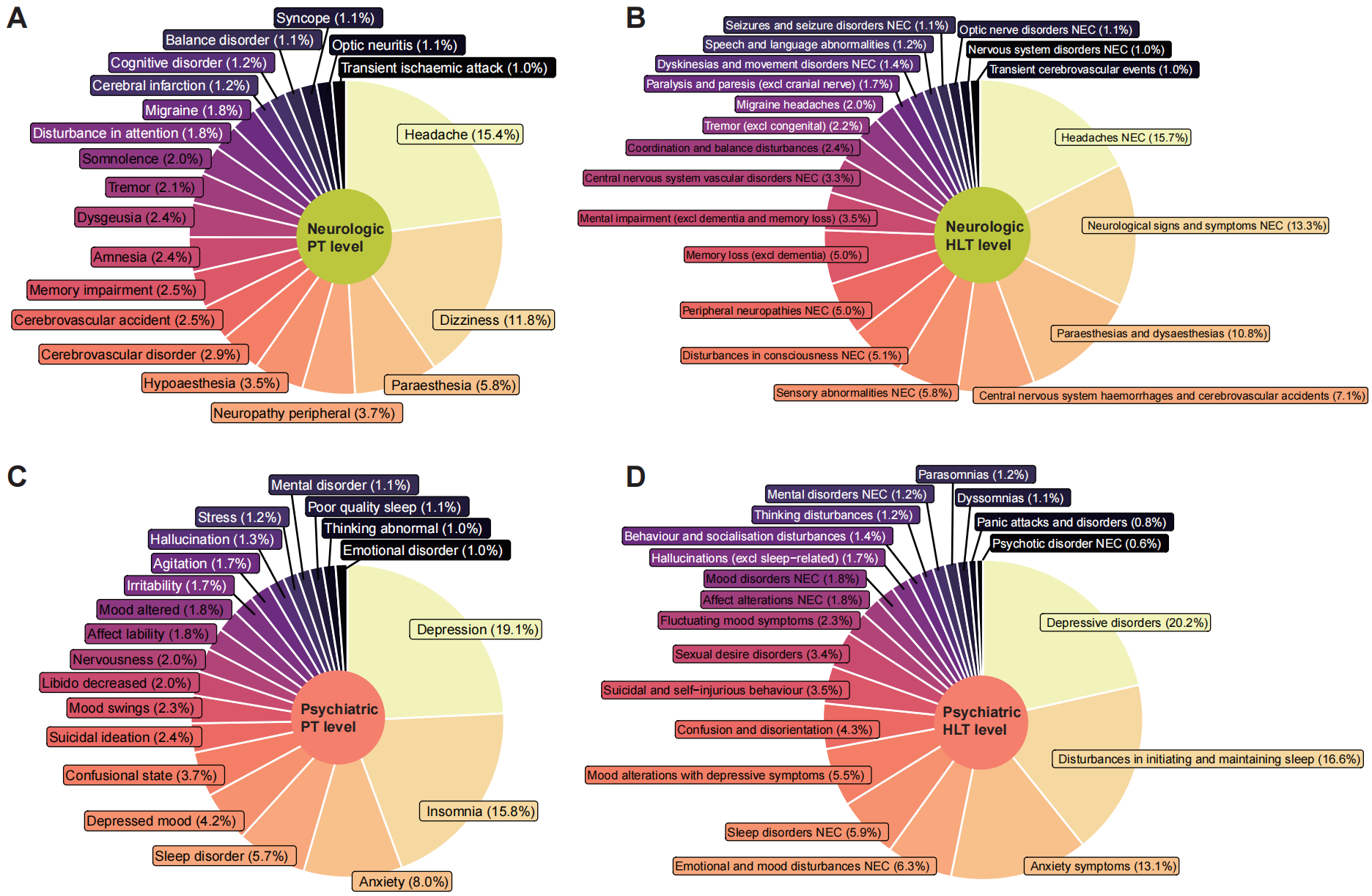


**Supplementary Figure 1.** Burden of neurologic and psychiatric events at the PT and HLT levels for selective estrogen receptor modulators in the VigiAccess database. (**A**) Top 20 neurologic events by report proportion at the PT level; (**B**) Top 20 neurologic events by report proportion at the HLT level; (**C**) Top 20 psychiatric events by report proportion at the PT level; (**D**) Top 20 psychiatric events by report proportion at the HLT level. PT, Preferred Term; HLT, High Level Term.


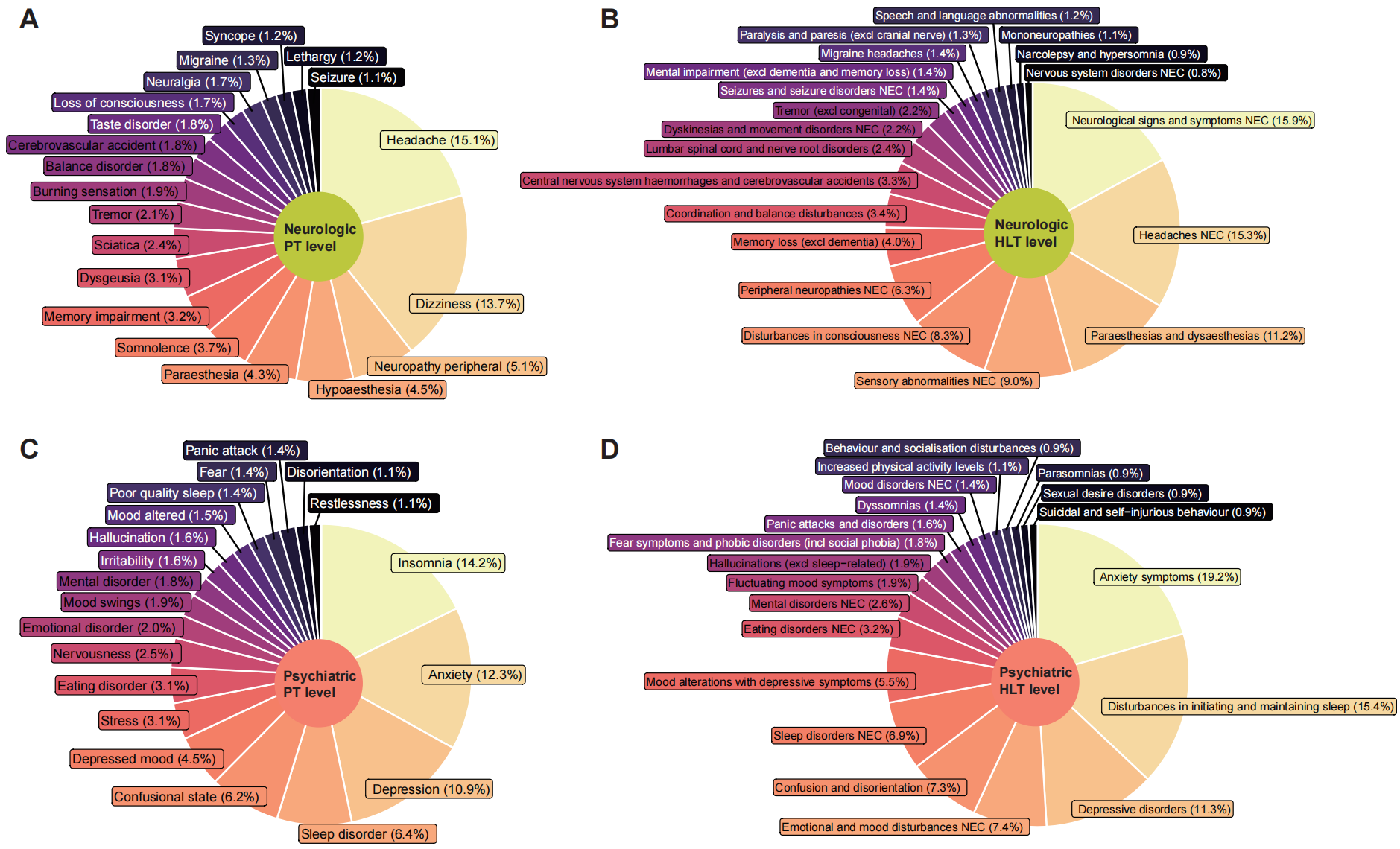


**Supplementary Figure 2.** Burden of neurologic and psychiatric events at the PT and HLT levels for selective estrogen receptor degraders in the VigiAccess database. (**A**) Top 20 neurologic events by report proportion at the PT level; (**B**) Top 20 neurologic events by report proportion at the HLT level; (**C**) Top 20 psychiatric events by report proportion at the PT level; (**D**) Top 20 psychiatric events by report proportion at the HLT level. PT, Preferred Term; HLT, High Level Term.


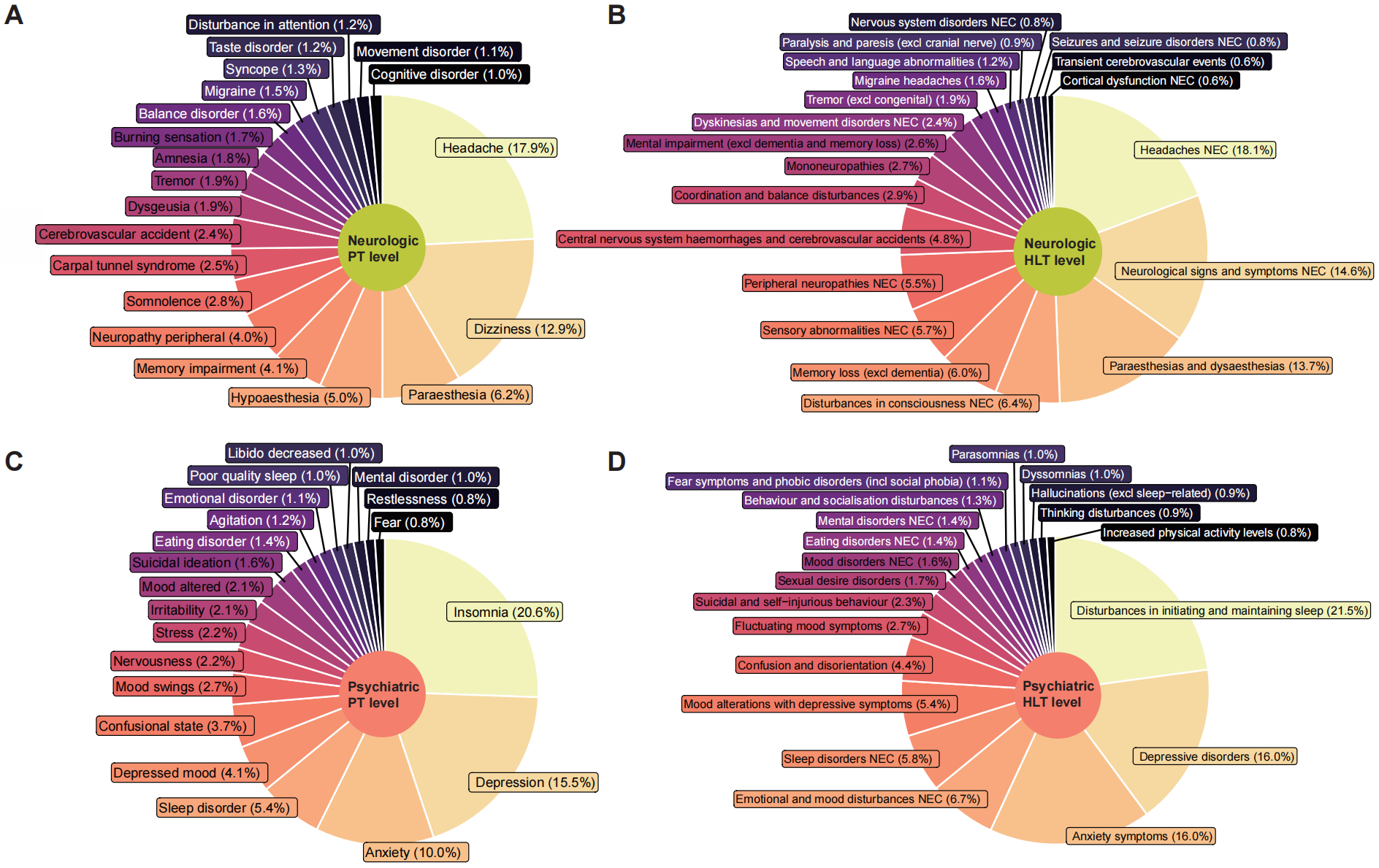


**Supplementary Figure 3.** Burden of neurologic and psychiatric events at the PT and HLT levels for aromatase inhibitors in the VigiAccess database. (**A**) Top 20 neurologic events by report proportion at the PT level; (**B**) Top 20 neurologic events by report proportion at the HLT level; (**C**) Top 20 psychiatric events by report proportion at the PT level; (**D**) Top 20 psychiatric events by report proportion at the HLT level. PT, Preferred Term; HLT, High Level Term.


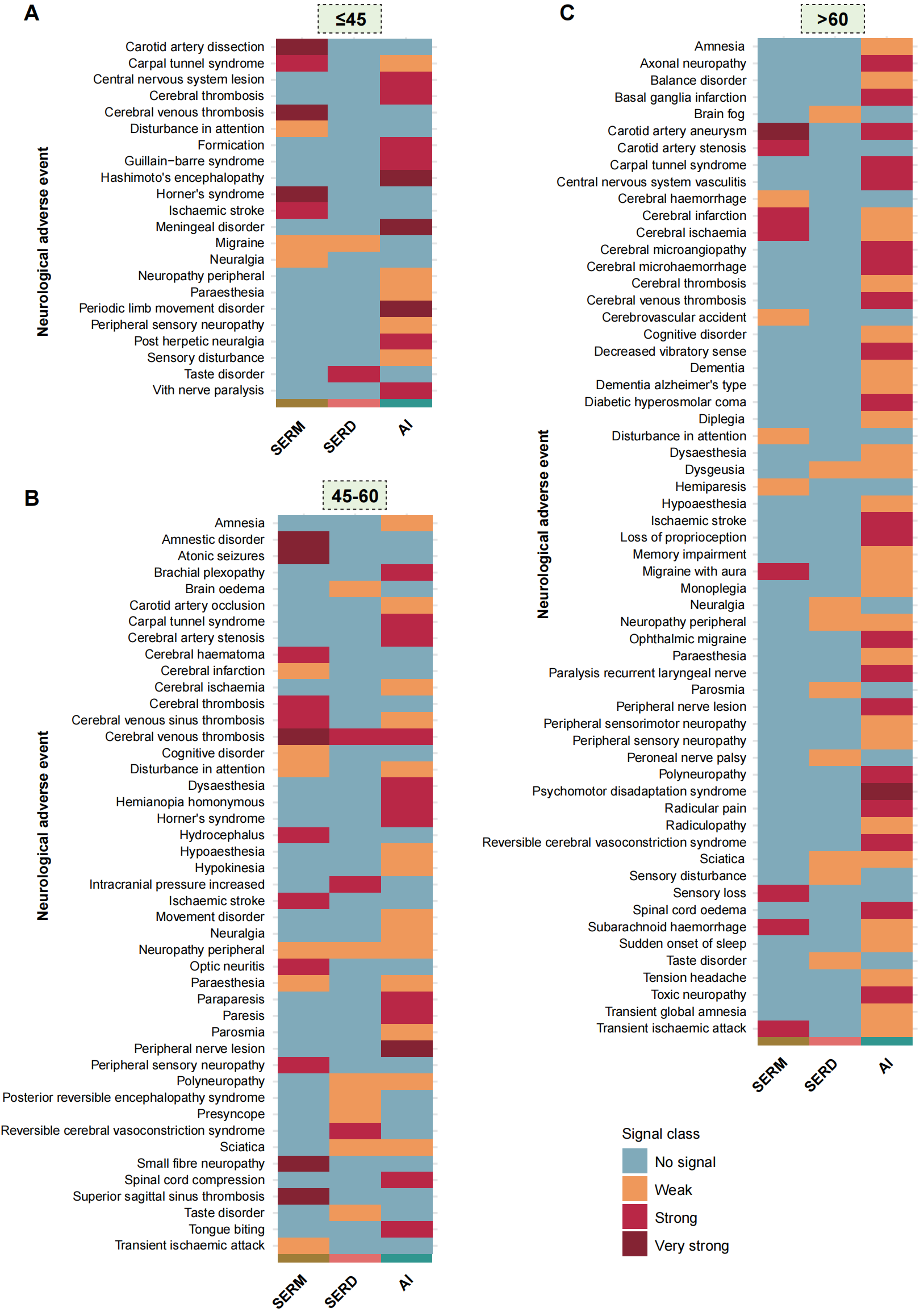


**Supplementary Figure 4.** Neurotoxicity safety signals of different breast cancer endocrine therapies by age groups. (**A**) ≤45 years old age group, (**B**) 45-60 years old age group, (**C**) >60 years old age group. Signal strength was classified by ROR magnitude: very strong (ROR > 30); strong (ROR between 5 and 30); and weak (ROR < 5). ETs, Endocrine Therapies. SERMs, selective estrogen receptor modulators; SERDs, selective estrogen receptor degraders; AIs, aromatase inhibitors.


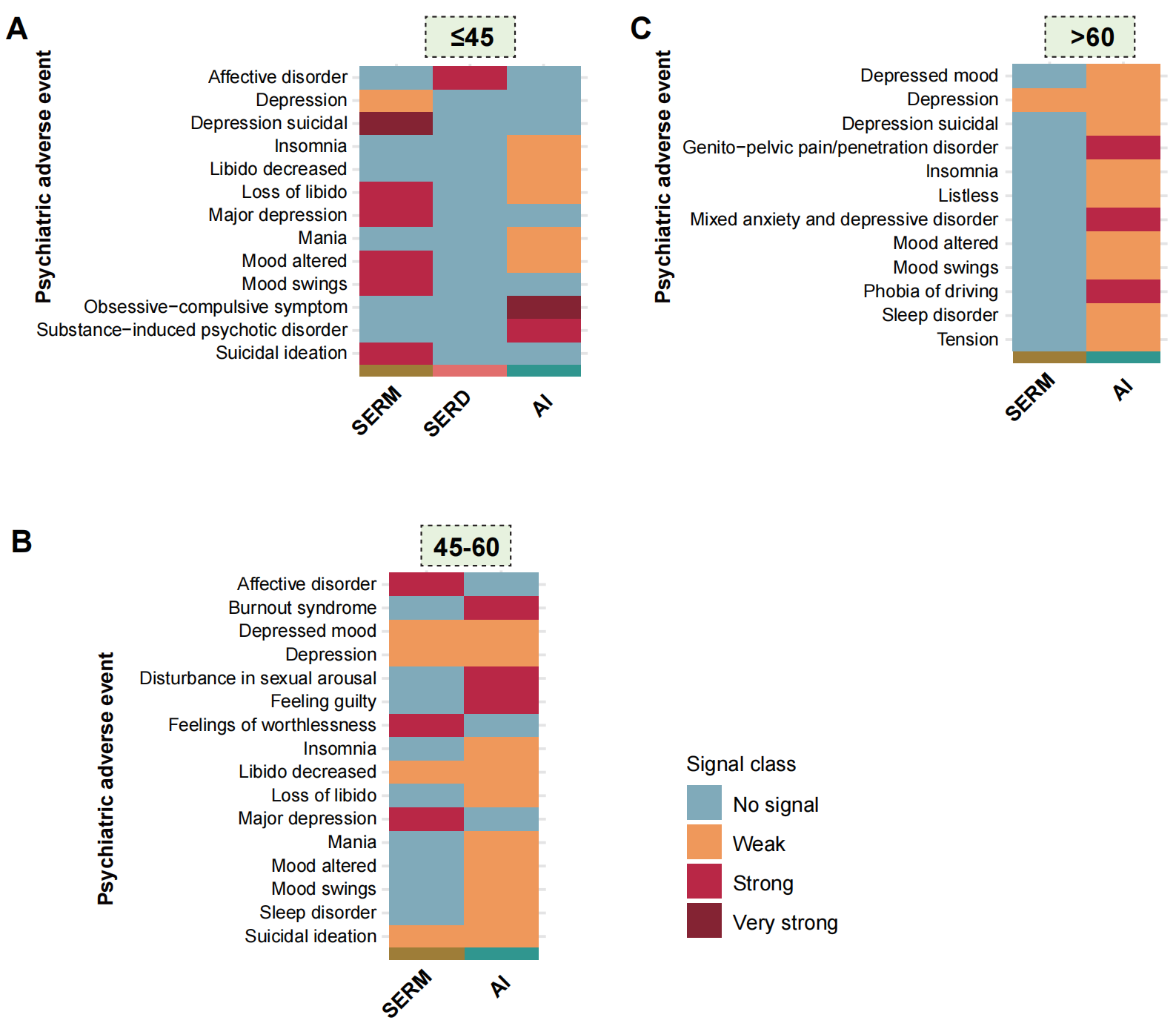


**Supplementary Figure 5.** Psychiatric safety signals of different breast cancer endocrine therapies by age groups. (**A**) ≤45 years old age group, (**B**) 45-60 years old age group, (**C**) >60 years old age group. Signal strength was classified by ROR magnitude: very strong (ROR > 30); strong (ROR between 5 and 30); and weak (ROR < 5). ETs, Endocrine Therapies. SERMs, selective estrogen receptor modulators; SERDs, selective estrogen receptor degraders; AIs, aromatase inhibitors.


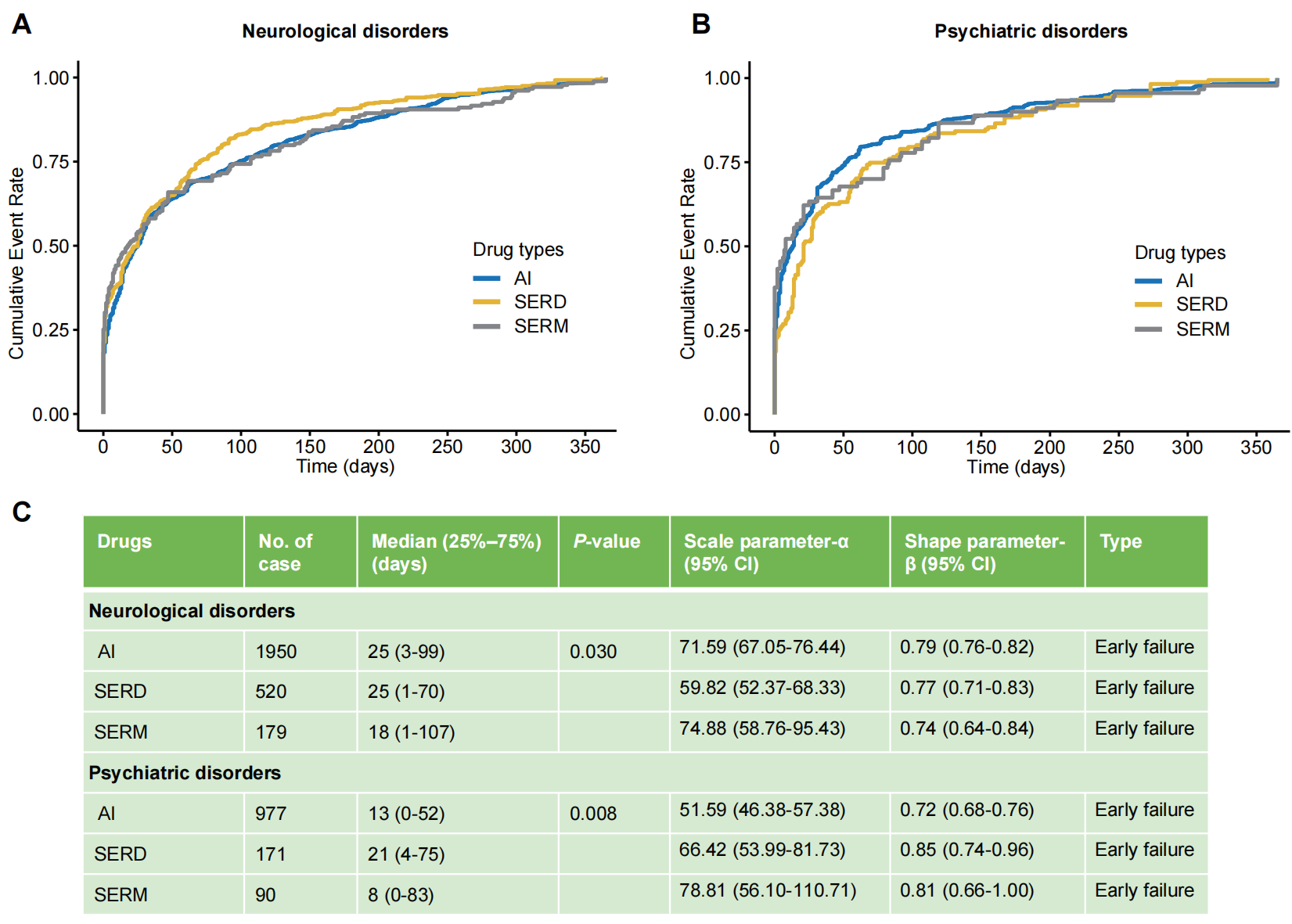


**Supplementary Figure 6.** Sensitivity analysis of time to onset for neurologic and psychiatric events across endocrine therapy classes after excluding reports with a time to onset longer than one year. (**A**) Cumulative pattern of neurologic events by time-to-onset; (**B**) Cumulative pattern of psychiatric events by time-to-onset; (**C**) Weibull distribution shape analysis of event onset patterns. SERDs, selective estrogen receptor degraders; SERMs, selective estrogen receptor modulators; AIs, aromatase inhibitors.
